# Supplementary material for: Defective transcription elongation in a subset of cancers confers immunotherapy resistance
Source: Nat Commun. 2018 Oct 23;9:4410. doi: 10.1038/s41467-018-06810-0 (PMC6199328; doi:10.1038/s41467-018-06810-0)
Supplement: Supplementary file 1 — Supplementary Information [file 41467_2018_6810_MOESM1_ESM.pdf]

Supplementary Information

Defective transcription elongation in a subset of cancers confers immunotherapy resistance

Modur et al.

Supplementary Figures

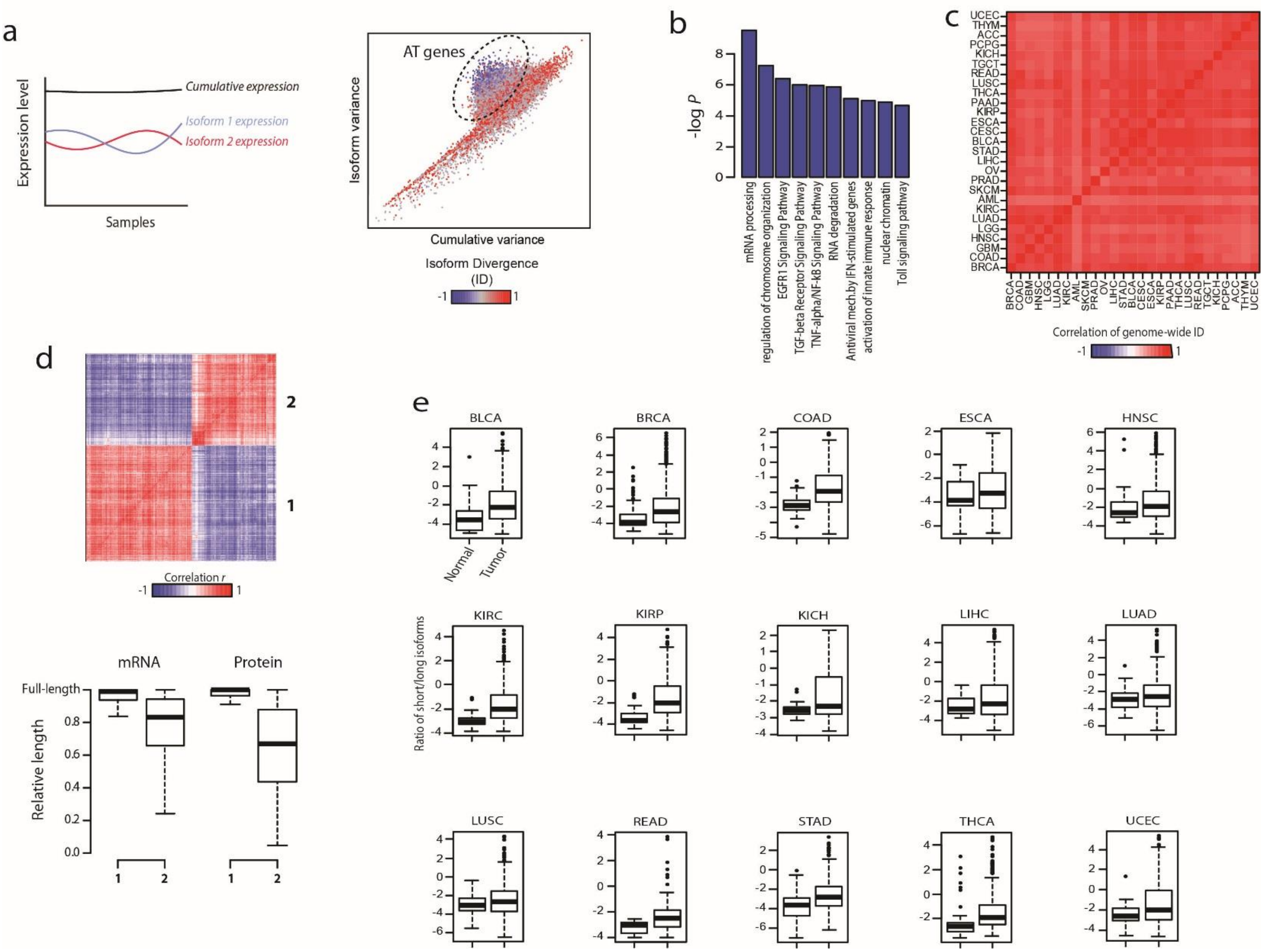

Supplementary Fig.1. A subset of cancers is characterized by widespread expression of truncated mRNAs of alternatively transcribed (AT) genes. a) Left: Expression characteristics of a gene at the level of its isoforms (red and blue lines) can be differentiated from its gene-level (cumulative) expression characteristics (black line). Right: each point is a gene, scattered by their cumulative or average isoform-level expression variance. Coloring of genes reflects the lowest pair-wise expression correlation (isoform divergence) of its isoforms, where negative correlation indicates isoform switching. The circled region contains candidate AT genes. The values were calculated using KIRC dataset. Other datasets gave near-identical distributions (not shown), b) correlation matrix of genome-wide isoform divergence values between different cancers, c) Pathway enrichment profile of the AT genes (hypergeometric distribution), d) top: pair-wise correlation matrix of expressions of the transcript isoforms of AT genes. To identify coherently regulated transcript clusters, only the transcript isoforms that have a correlation less than -0.5 ($r < -0.5$) with at least 5 other transcripts were included in this matrix. Two mutually exclusive clusters of isoforms (1 and 2) are indicated, bottom: mRNA and predicted protein lengths (relative to full-length) of the transcript isoform clusters 1 and 2, e) log ratios of average expression of short to full-length isoforms for AT genes in tumor and adjacent normal samples in the indicated cancers. Several cancer types (e.g. GBM, LGG and SKCM) did not have adjacent normal tissue samples. Boxplots: middle line: median, boxed areas extend from the first to third quartile; whiskers show 1.5 x inter-quartile range from the first (bottom) or third (top) quartile.

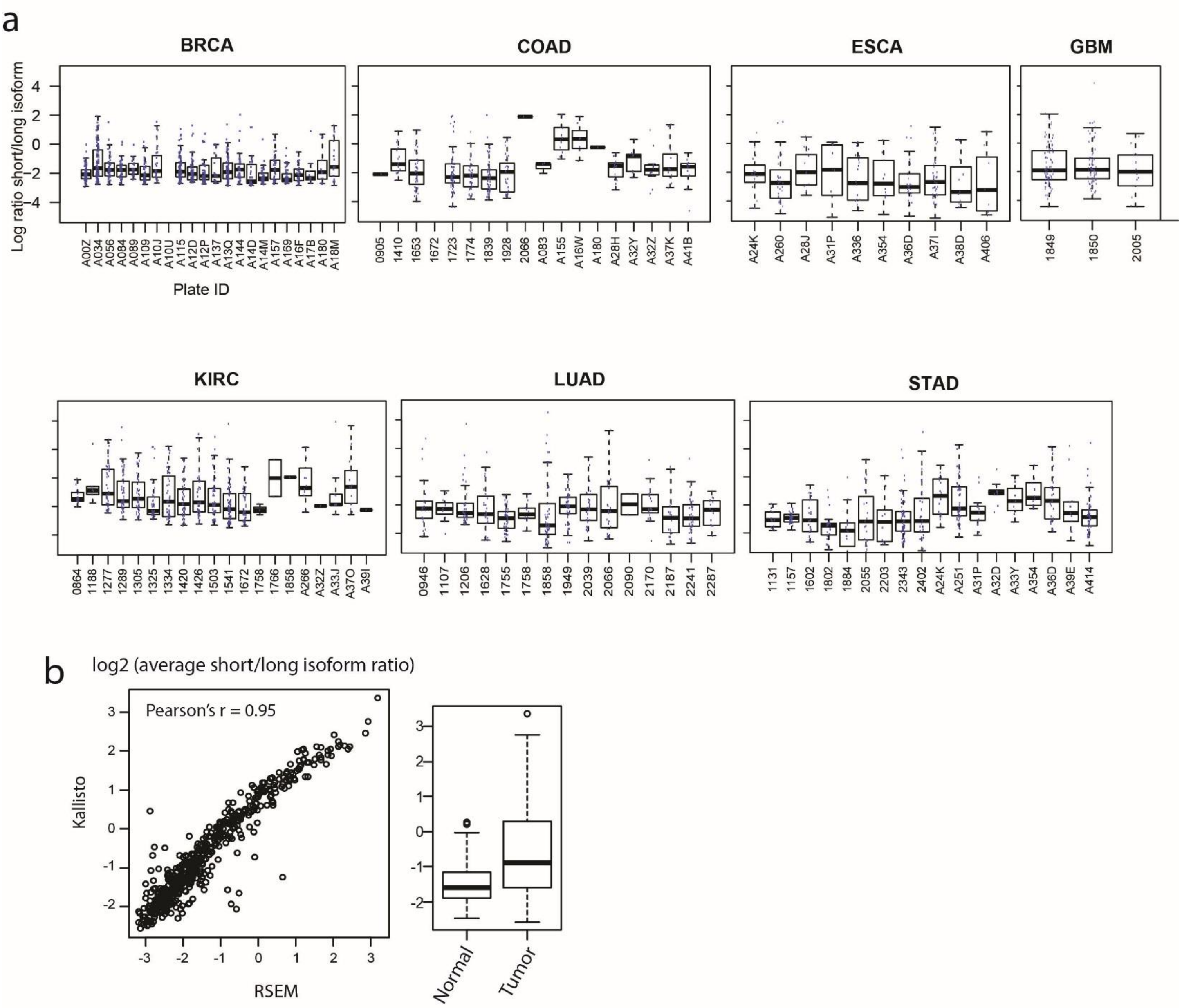

Supplementary Fig.2. a) Ratio of average relative expression of short to long isoforms for AT genes stratified by RNAseq batches (plate ID) in the indicated cancer datasets, b) left: correlation of ratio of short to long isoform expression calculated by two different quantitation methods: RSEM and Kallisto; right: ratio of short to long isoform expression quantified by Kallisto in normal and cancer tissue for KIRC.

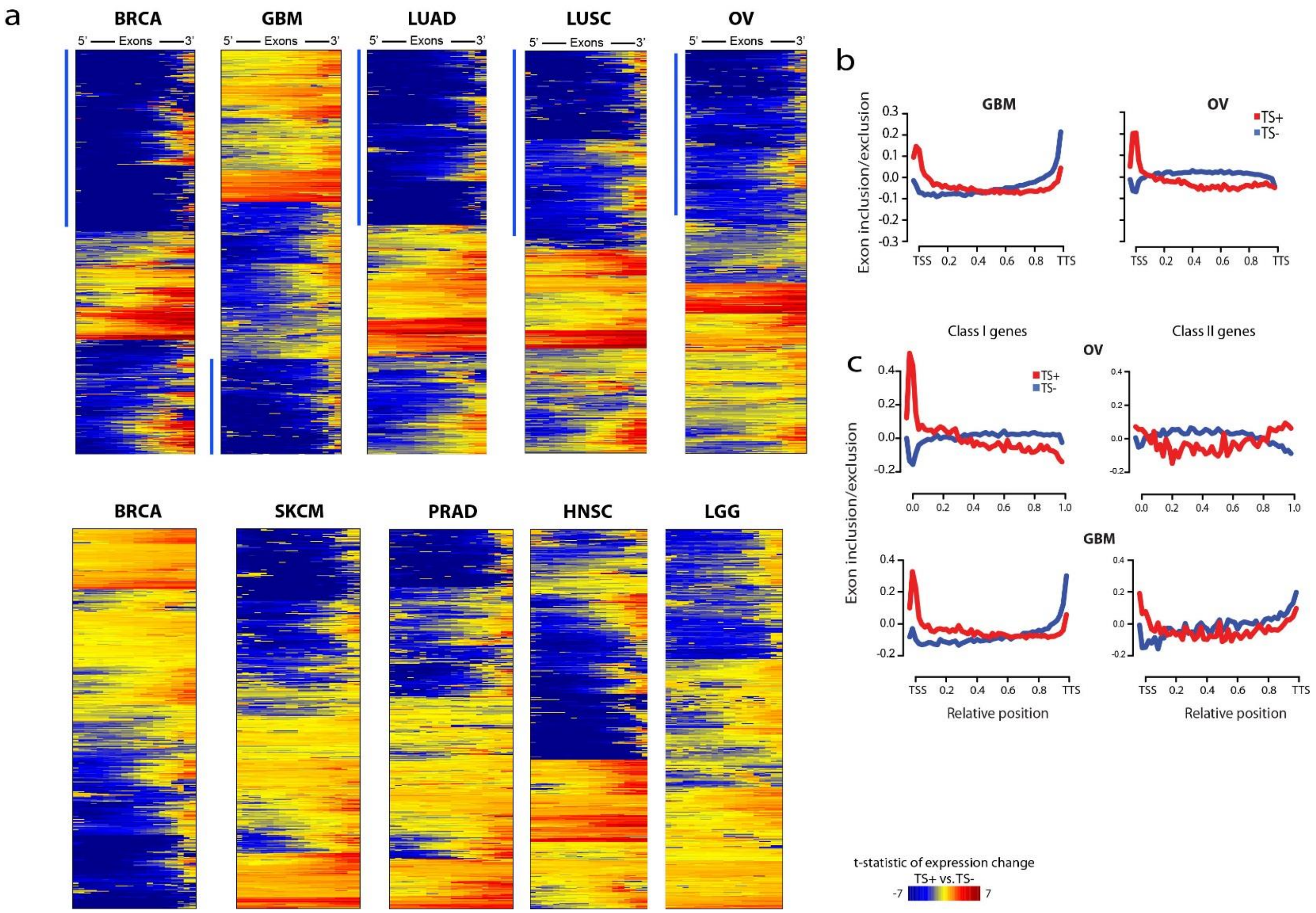

Supplementary Fig.3. a) Differential (TE^{deff} vs. TE^{prof} samples) exon expression heatmaps for the indicated cancers (compare with Fig.1c), b) Genome-wide exon inclusion/exclusion profiles of genes according to the gene body position based on Affymetrix Exon array data (same as in Fig.1e) for GBM and OV datasets for all and (c) and class I or class II genes.

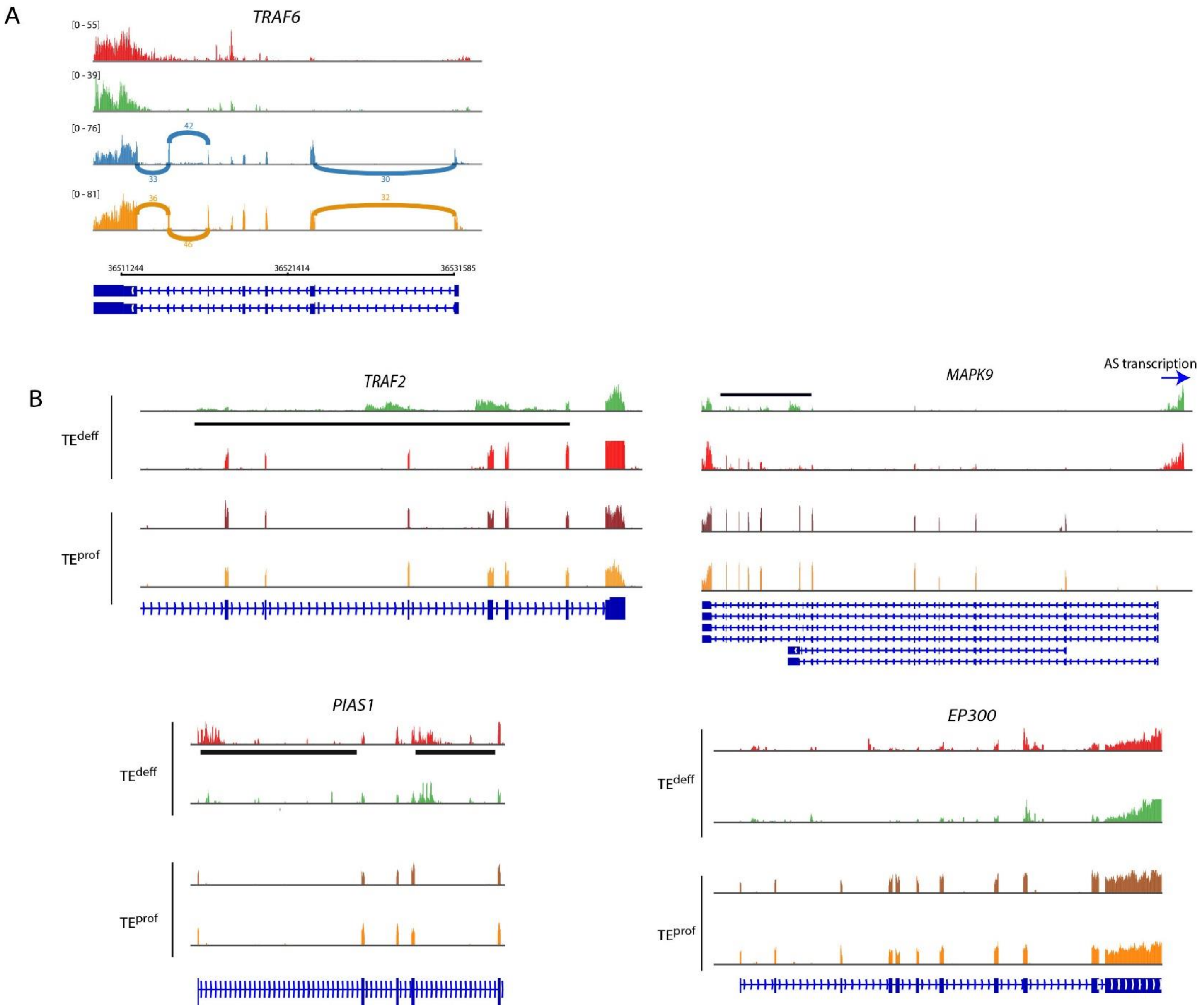

Supplementary Fig.4. Intronic and spurious transcription in samples with TE^{deff}. Exon and intron coverage of RNAseq reads of 2 representative TE^{deff} and TE^{prof} samples from portions of indicated genes. A) Whole gene for *TRAF6* is shown, related to Fig.1H. B) Some of other representative genes. Only portions of the genes are shown for clarity. Note extensive mapping of reads to intronic and exon-intron boundary regions in TE^{deff} samples. Also note extensive antisense transcription from the TSS in *MAPK9* gene in the TE^{deff} samples.

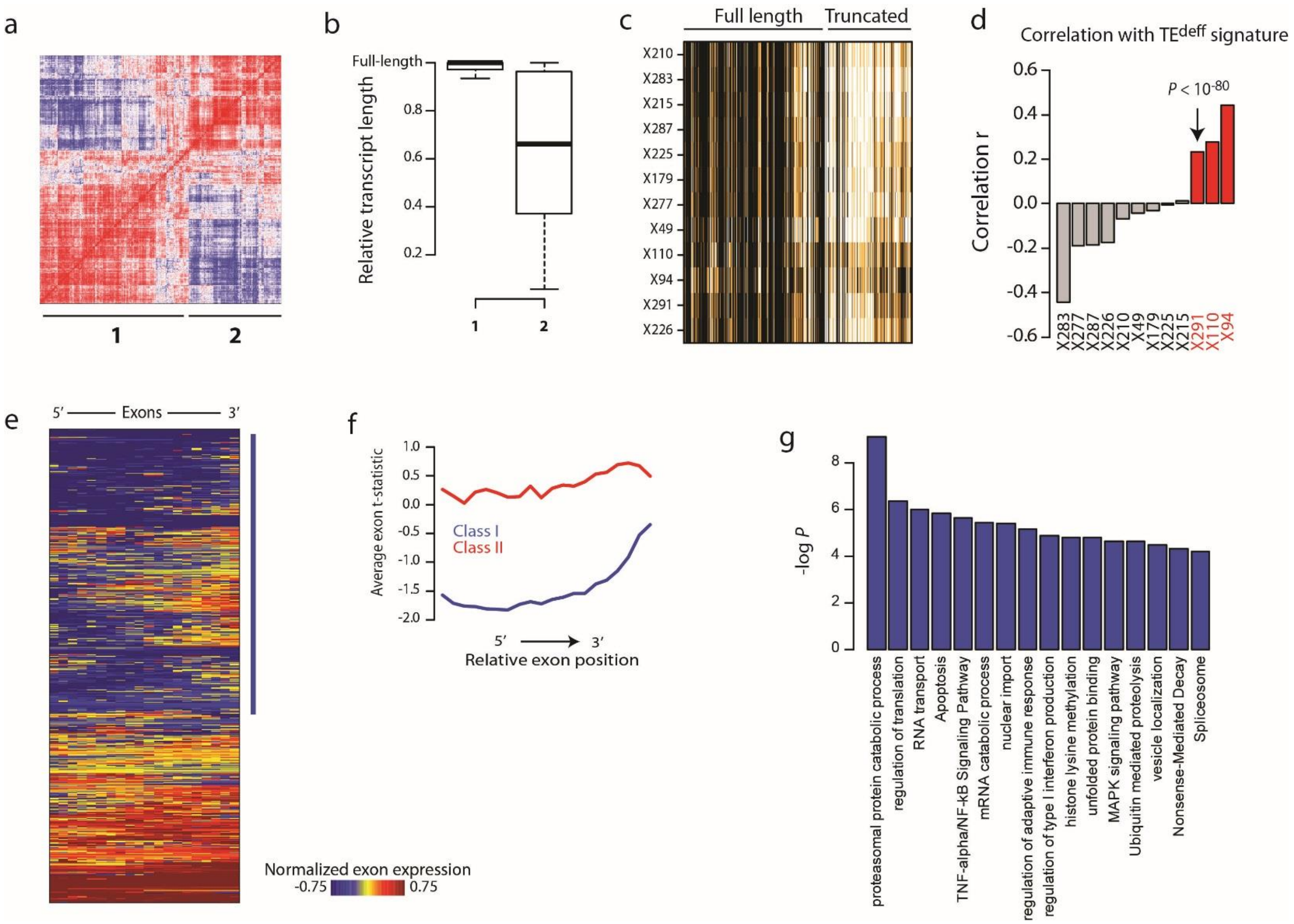

Supplementary Fig.5. TE^{deff} can be identified in an independent renal cell carcinoma tissue cohort of 12 samples, a) Correlation matrix of isoforms of AT genes in this RCC cohort, b) relative lengths of the clusters in 1 and 2 in (a), c) Relative isoform expression matrix of full-length (cluster 1) and truncated (cluster 2) isoforms (see (b)) in the 12 RCC samples, d) Correlation of gene-level transcriptomic signatures of indicated RCC samples with the average LTF signature from TCGA. The three potential LTF+ samples are highlighted. The p-value shown is for the least significant TE^{deff} sample, e) differential exon expression heatmap for a TE^{deff} sample (X94) with spuriously transcribed genes (potentially class I) highlighted with blue line, f) Median exon t-statistic of difference (TE^{deff} vs. TE^{prof} samples in this RCC cohort) of class I and II genes (same as in Fig.1d for TCGA KIRC cohort), g) pathway enrichment profile of genes highlighted in (e). Note similarity with the profile in Fig.1h for class I genes from TCGA KIRC samples.

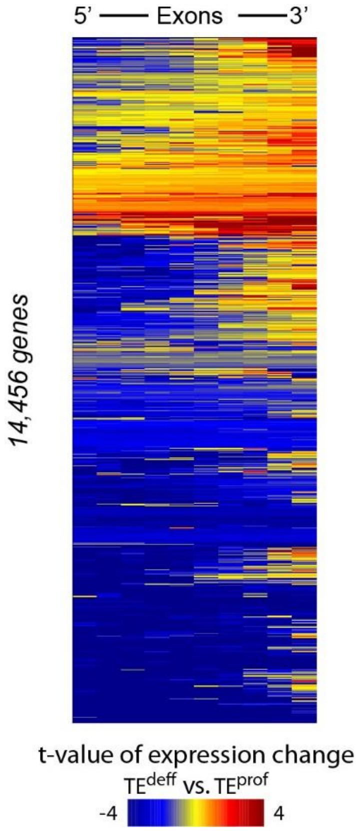

Supplementary Fig.6. Differential exon expression heatmap (same as in Fig.1c) for TE^{deff} (n = 2) vs. TE^{prof} (n = 52) breast cancer cell lines based on RNAseq data for 54 breast cancer cell lines in CCLE.

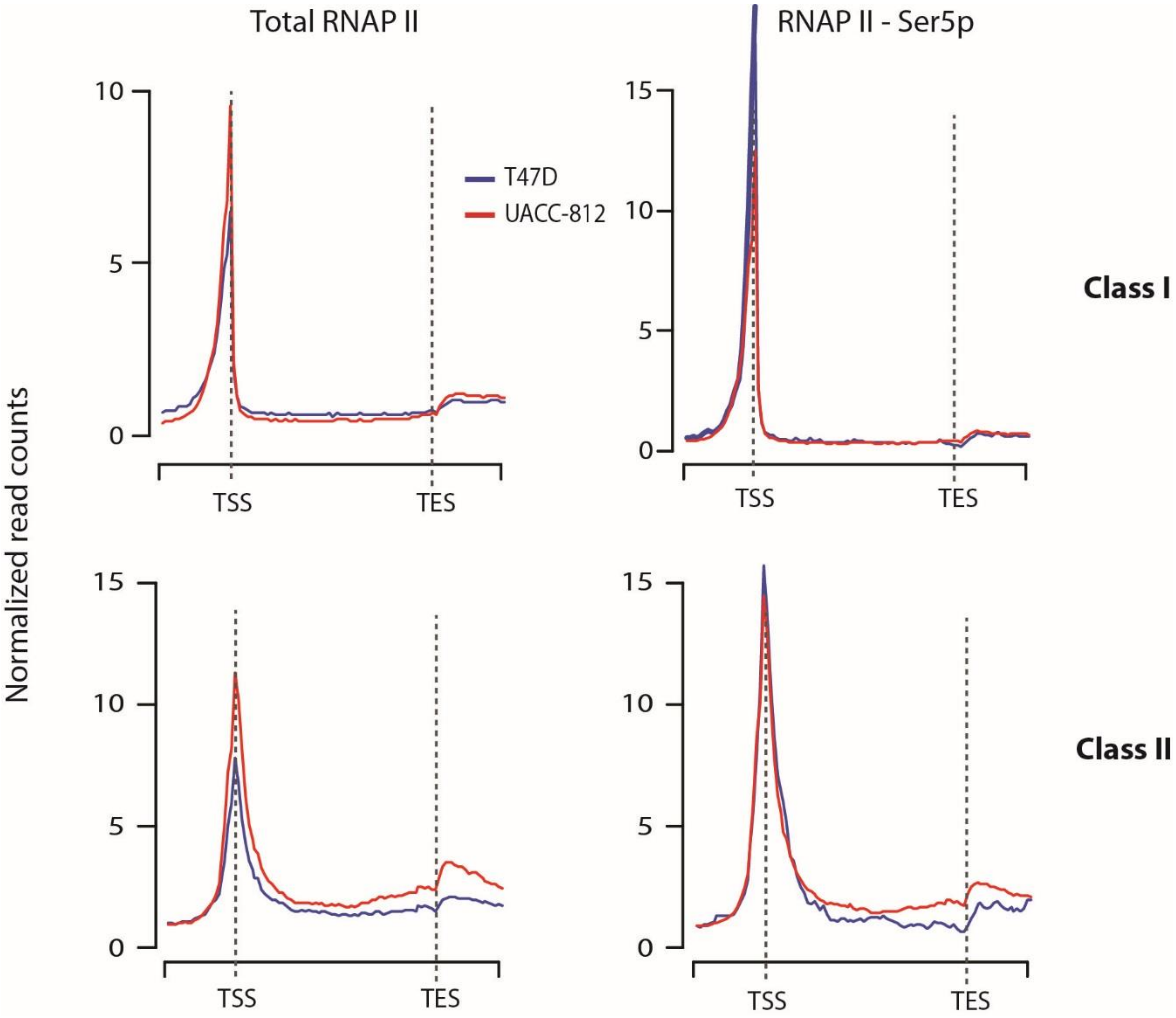

Supplementary Fig.7. Gene body occupancy profiles of total (left) and Ser5-phosphorylated (right) RNAP II in a TE^{deff} (UACC-812) and TE^{prof} (T47D) line in Class I (top) and Class II (bottom) genes. Based on ChIP-seq experiments.

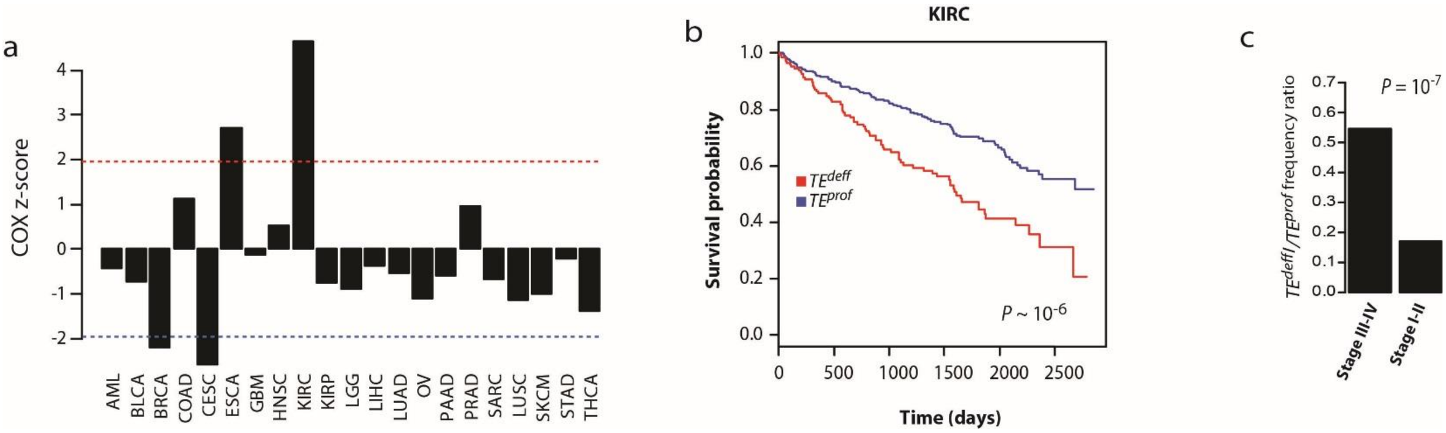

Supplementary Fig.8. a) Pan-cancer analysis of correlation of TE^{deff} with overall survival. Z-scores from COX proportional hazards regression analysis are shown on the y-axis. Cutoff points corresponding to $P \sim 0.05$ are indicated by horizontal lines. b) Kaplan-meier survival curves for overall survival in TE^{deff} and TE^{prof} samples in KIRC, c) Relative frequencies of TE^{deff} and TE^{prof} samples in early- and late-stage KIRC. The p-value shows Fisher’s exact test.

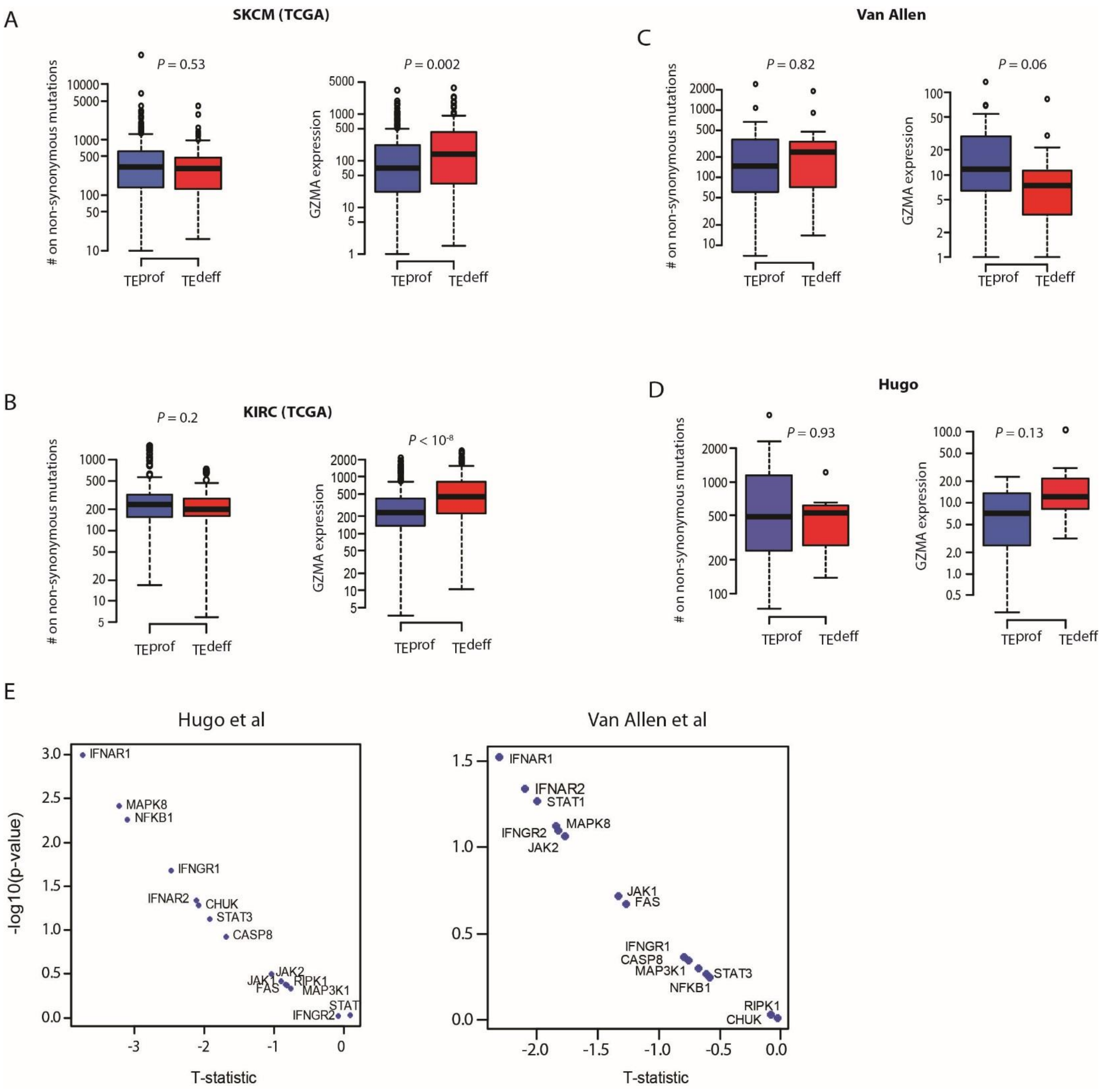

Supplementary Fig.9. Comparative analysis of the non-synonymous mutational load and cytolytic T cell infiltration (GZMA expression in the bulk tumor) in TE^{deff} and TE^{prof} tumors in the SKCM and KIRC cohorts in TCGA (A-B), and in the Van Allen et al (C) and in the Hugo et al (D) cohorts. *P*-values reflect Wilcoxon rank sum test. E) T-statistic by p-value (-log10) plot of expression difference for the indicated immunotherapy-relevant genes in TE^{deff} vs TE^{prof} samples in the Van Hugo et al and Van Allen et al datasets.

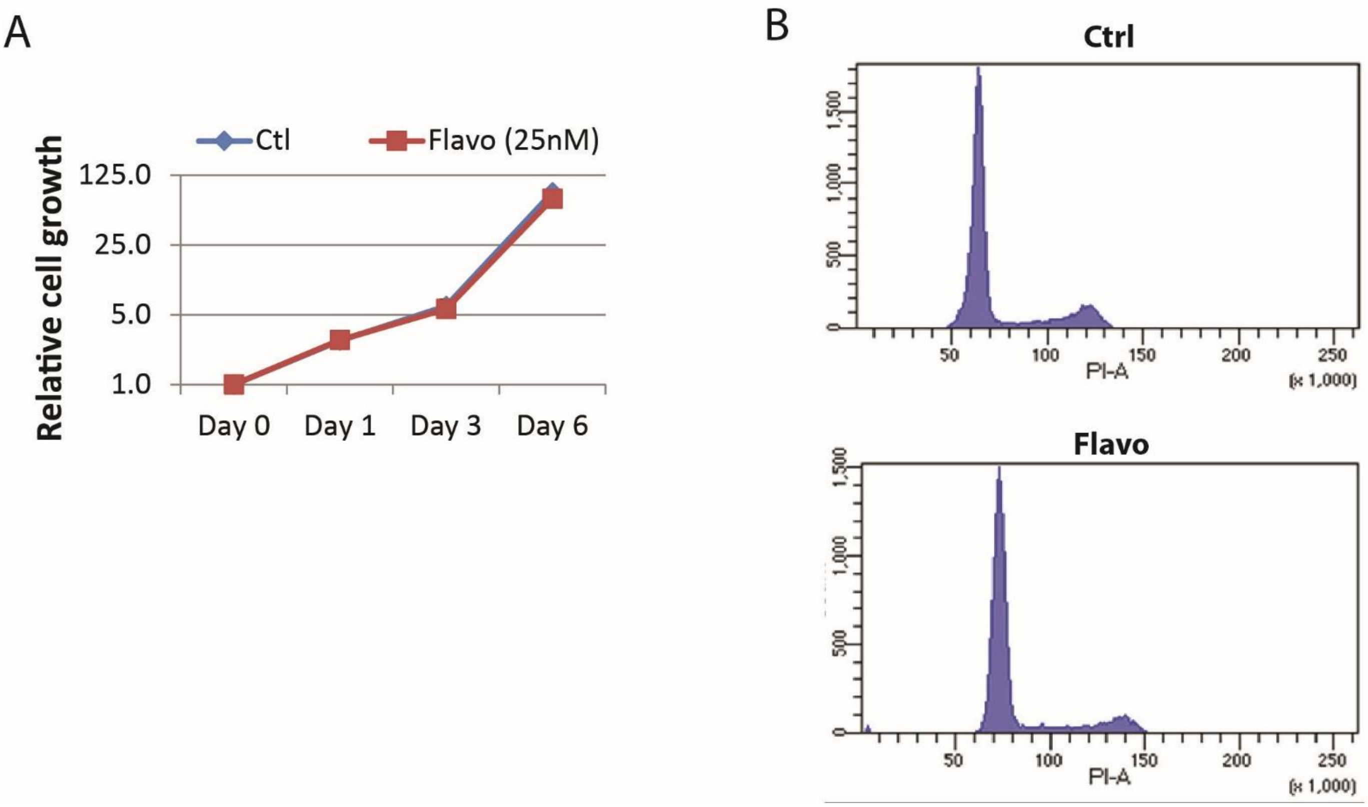

Supplementary Fig.10. Cell growth characteristics of B16F10 cells chronically treated with low-dose flavopiridol. A) Viability (measured by PrestoBlue) of control and flavopiridol-treated cells B16F10 at indicated days post-treatment. B) Cell cycle profiles (propidium iodide staining) of control and 7-day flavopiridol-treated cells.

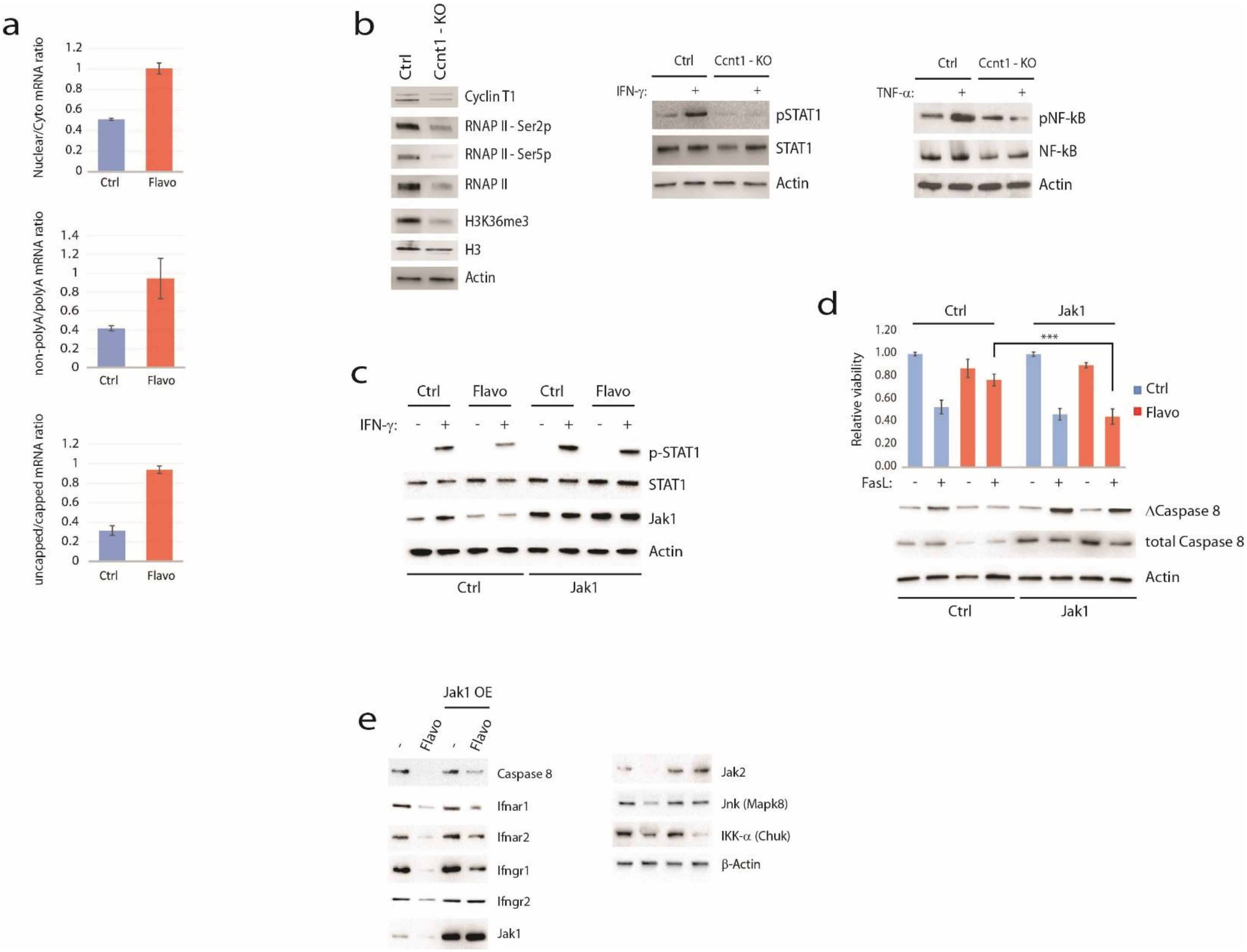

Supplementary Fig.11. Chronic inhibition of CDK9 by flavopiridol recapitulates TE^{deff}-like molecular characteristics, a) nuclear/cytoplasmic, non-polyA-ed/polyA-ed and uncapped/capped mRNA ratios in control and chronically flavopiridol-treated B16F10 cells, b) Immunoblots of the parental and Ccnt1-KO CT26 cells for the indicated proteins. Left: Blots of RNAP II and elongation markers, middle: blot of STAT1 phosphorylation after IFN-γ treatment, and right: blot of NF-κB phosphorylation after TNF-α treatment, c) Control and Jak1-overexpressing CT26 cells were treated with IFN-γ for 30 minutes, and the lysates were blotted with indicated markers, d) Relative viability (top) and caspase 8 activity measured by western blot (bottom) of control and Jak1-overexpressing CT26 cells after treatment with FasL. ***: $P < 0.001$, e) western blots of the indicated inflammatory response pathway proteins in the control and Jak1-overexpressing (Jak1 OE) CT26 cells before and after chronic flavopiridol treatment. Barplots: standard deviation of 3 replicates.

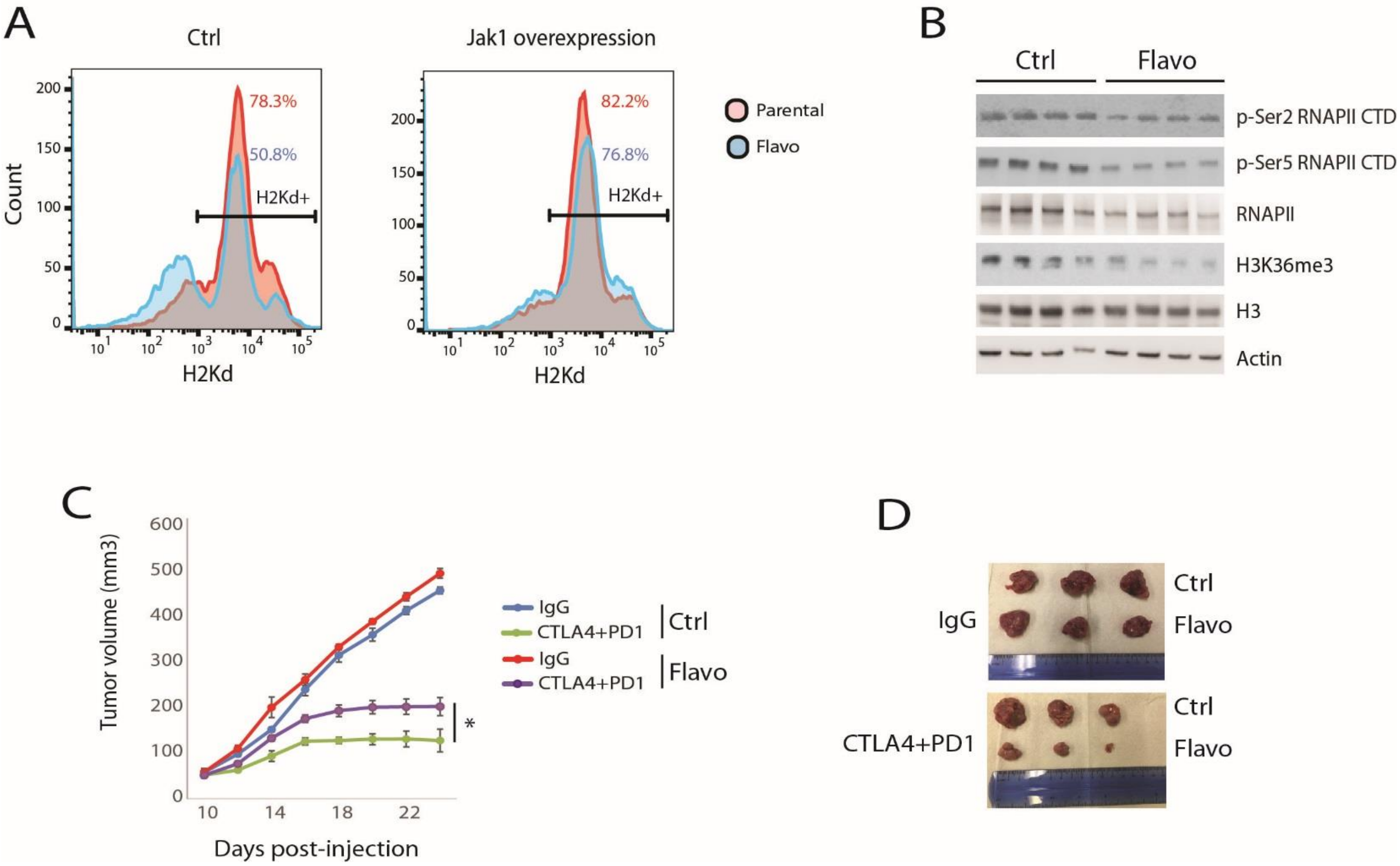

Supplementary Fig.12. A) Flow cytometry-based quantitation of cell surface H2-K^{D} expression in the control and Jak1-overexpressing CT26 cells before and after chronic flavopiridol treatment. The numbers show percentages of H2-K^{D}+ cells in the respective samples (red: parental, blue: flavo), b) western blots of tumors from control and flavopiridol pre-treated cells in the end of the experiment in Fig.7E were excised and blotted with the RNAP II and H3K36me3 antibodies as shown. Note persistence of transcriptional defects in flavo pre-treated cells 3 weeks after drug release and injection into mice, c-d) Same as in Fig.7F-G, but mice were treated with a combination of anti-CTLA4 and –PD1 antibodies.

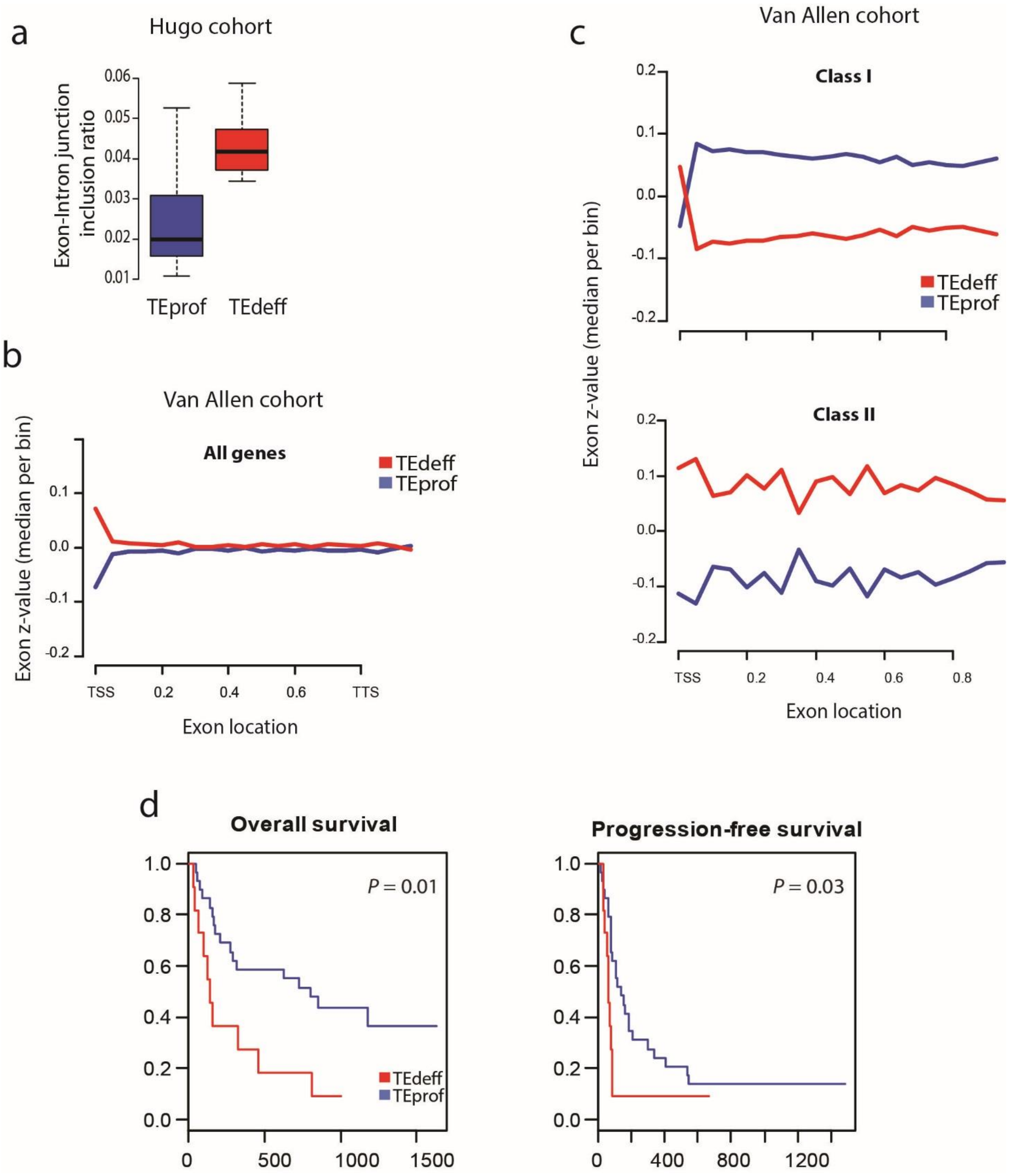

Supplementary Fig.13. Definition of TE^{deff} in the Hugo and Van Allen cohorts and associated markers, a) genome-wide exon-intron junction retention in class I genes in TE^{deff} and TE^{prof} samples in the Hugo cohort, b) Exon expression z-value (z-normalized across samples) of genes stratified by their relative position along the gene, in TE^{deff} and TE^{prof} samples in the Van Allen dataset. The Van Allen dataset is non-polyA-selected dataset (total mRNAseq), therefore the exon expression profile reflects that from Affymetrix Exon arrays from TCGA (see Fig.1F), c) Same as in (b), but for Class I and Class II genes. The mirroring effect of TE^{deff} and TE^{prof} cases in (b) and (c) is due to the z-score normalization of equal numbers of TE^{deff} and TE^{prof} cases in the Van Allen cohort, d) TE^{deff} samples in the Van Allen cohort were defined as the upper 25% of exon-intron junction retention values (The TE^{deff} samples in this cohort in Fig.4D and Supplementary Fig.8 were based on the cutoff at the median). The survival curves for overall and progression-free survival of resultant TE^{deff} and TE^{prof} samples in this cohort.

Supplementary Fig.14. Raw images of key blots presented in the manuscript. Molecular weight of the closes MW markers are indicated. Corresponding figure panels for the images are indicated on top left corner. Note that some membranes were cropped prior to blotting to incorporate more antibodies.

Fig 3G

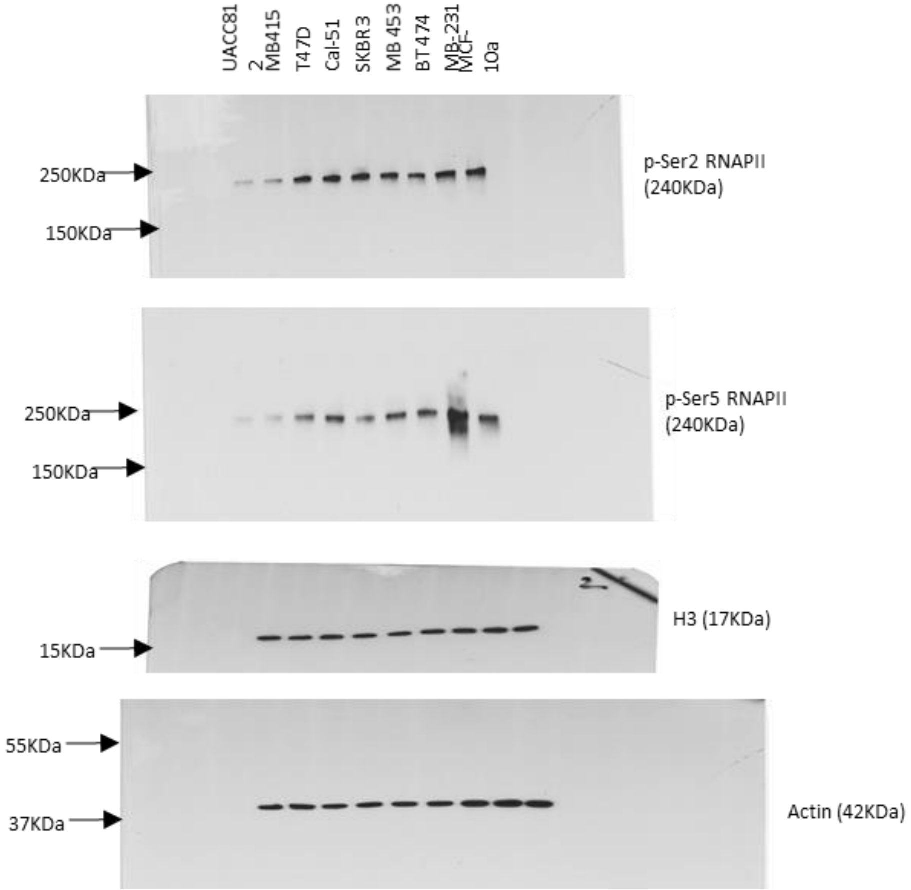

Fig 5D

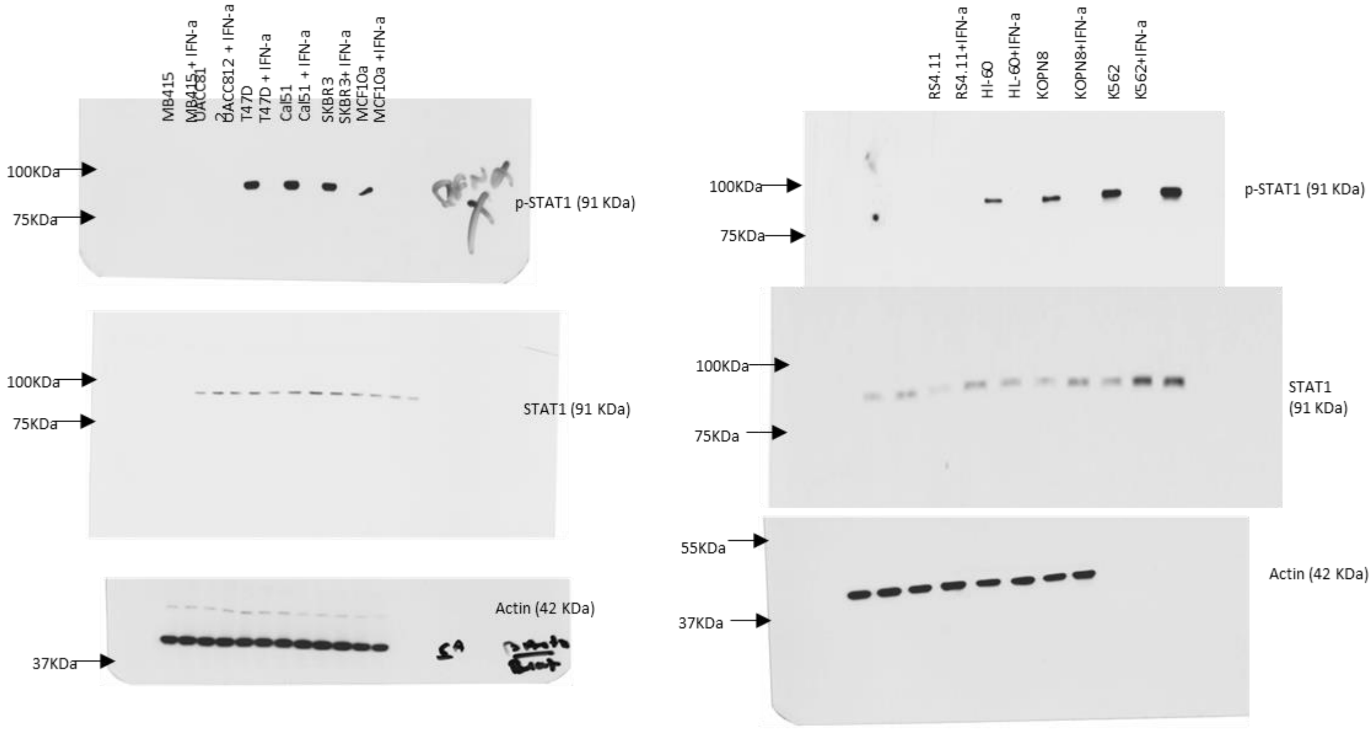

Fig 5D

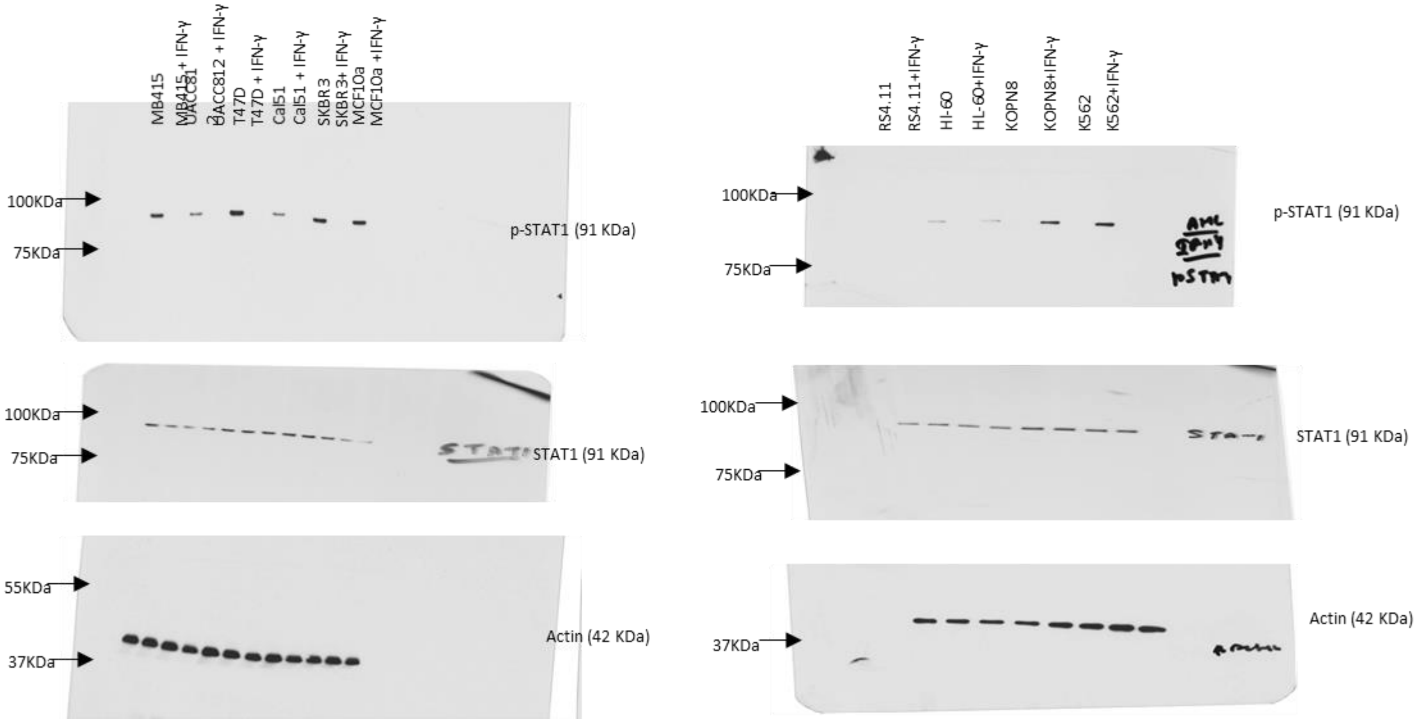
MB415
MB415 + IFN-γ
UACC812
UACC812 + IFN-γ
T47D
T47D + IFN-γ
Cal51
Cal51 + IFN-γ
SKBR3
SKBR3+ IFN-γ
MCF10a
MCF10a +IFN-γ
100KDa
75KDa
p-STAT1 (91 KDa)
STAT1 (91 KDa)
55KDa
37KDa
Actin (42 KDa)
RS4.11
RS4.11+IFN-γ
HI-60
HL-60+IFN-γ
KOPN8
KOPN8+IFN-γ
K562
K562+IFN-γ
100KDa
75KDa
p-STAT1 (91 KDa)
STAT1 (91 KDa)
37KDa
Actin (42 KDa)

Fig 5D

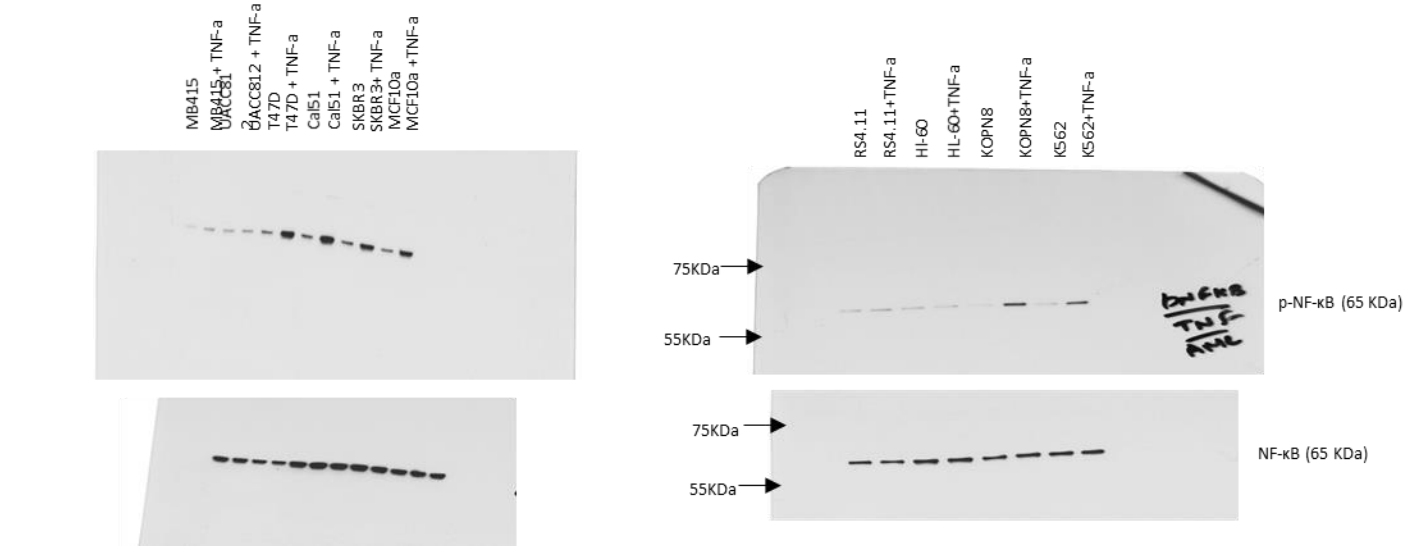
MB415
MB415 + TNF-a
UACC812
UACC812 + TNF-a
T47D
T47D + TNF-a
Cal51
Cal51 + TNF-a
SKBR3
SKBR3+ TNF-a
MCF10a
MCF10a +TNF-a
RS4.11
RS4.11+TNF-a
HI-60
HL-60+TNF-a
KOPN8
KOPN8+TNF-a
K562
K562+TNF-a
75KDa
55KDa
p-NF-κB (65 KDa)
NF-κB (65 KDa)

Fig 5G

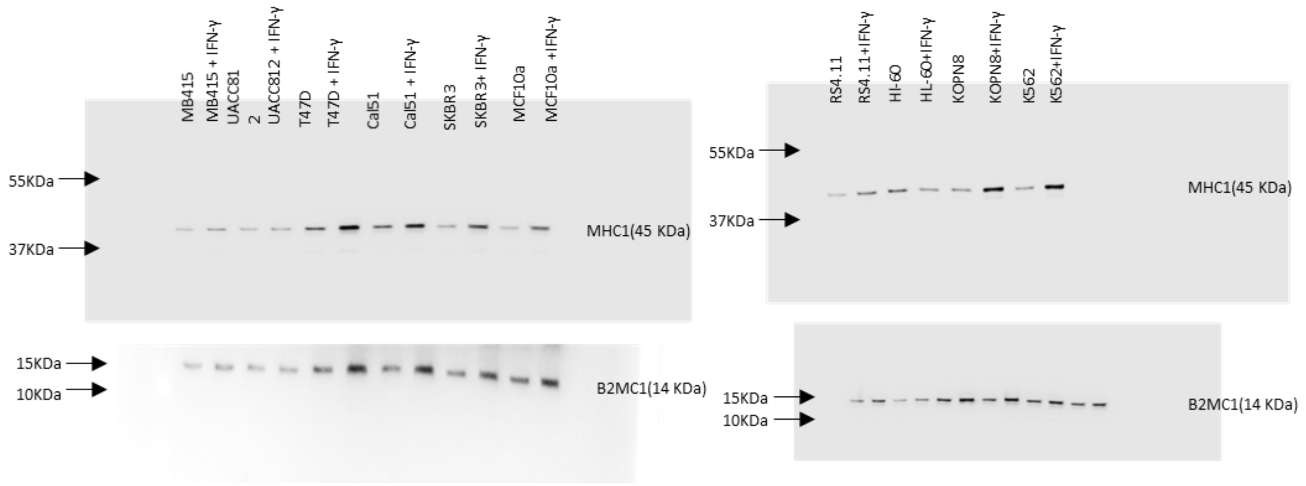

Fig. 6A

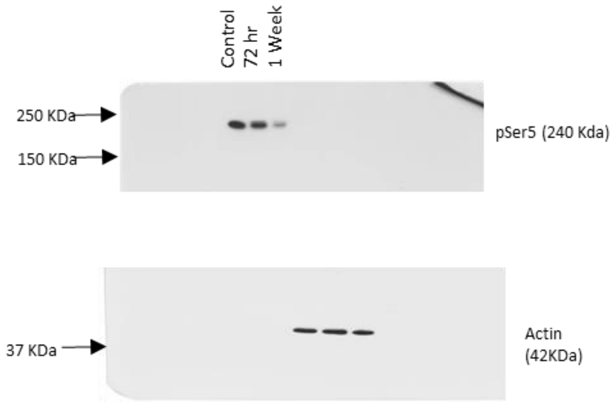

Fig 7A

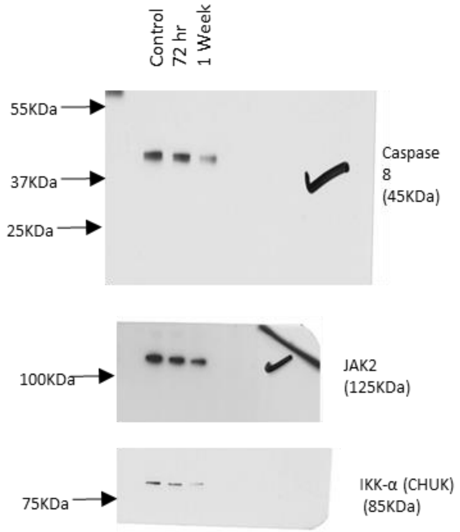

Fig.7H

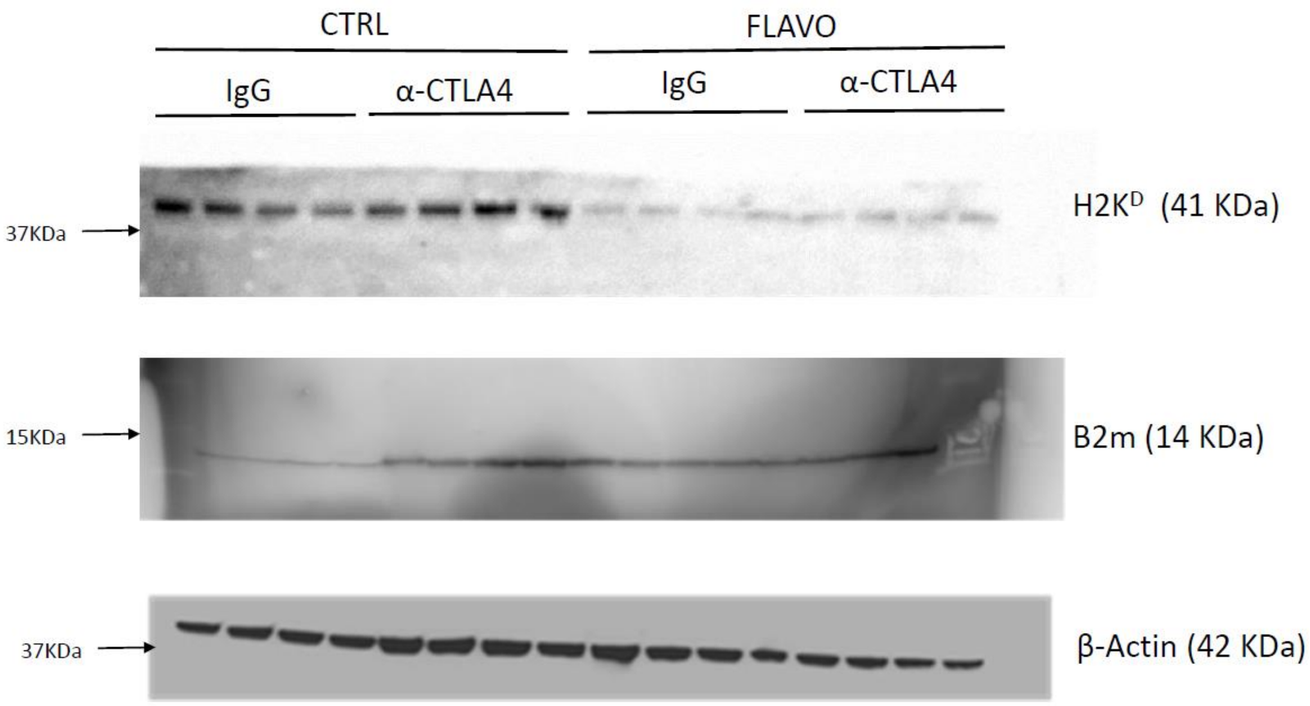
CTRL
FLAVO
IgG
α-CTLA4
IgG
α-CTLA4
37KDa
H2K^D (41 KDa)
15KDa
B2m (14 KDa)
37KDa
β-Actin (42 KDa)

Supplementary Tables

Supplementary Table 1. The TCGA datasets and the number of clinical, RNAseq and RPPA samples in each.

Cancer Type	Clinical	RNAseq	RPPA
BLCA	412	407	344
BRCA	1097	1093	887
COAD	459	285	362
CESC	307	304	173
ESCA	185	184	126
GBM	596	169	233
HNSC	528	520	212
KIRC	537	534	478
KIRP	291	290	216
LAML	200	173	-
LGG	515	534	429
LIHC	377	374	184
LUAD	522	515	365
OV	587	309	443
PAAD	185	178	123
PRAD	500	498	352
SARC	261	259	226
LUSC	504	501	328
SKCM	470	473	355
STAD	443	415	357
THCA	507	501	224
UCEC	548	176	440
READ	170	94	131
TGCT	134	150	122
KICH	113	66	63
PCPG	179	179	82
ACC	92	79	46
THYM	124	120	90

Supplementary Table 2. Class I and II genes from Fig.1D.

Class I	Class II
AASDHPPT	AAAS
ABCA1	AARSD1
ABCB10	ABCB8
ABCB7	ABCC10
ABCC1	ABHD11
ABCC4	ABHD14A
ABCD3	ACAP1
ABCE1	ACBD4
ABCF1	ACCS
ABHD2	ACOT8
ABI1	ACTR5
ABI2	ADRM1
ABL1	AGPAT2
ABLIM1	AIFM2
ACADM	AIP
ACADSB	AKR1A1
ACAP2	ALG3
ACBD3	ALKBH2
ACBD5	ALKBH6
ACLY	ALKBH7
ACOX1	ANAPC11
ACSL3	ANKRD13D
ACSL4	ANKRD37
ACTR2	ANKRD39
ACTR3	ANKRD54
ACVR1	ANKS3
ACVR1B	AP2S1
ADAM10	AP4M1
ADAM17	APBA3
ADAM9	APBB3
ADAMTSL3	APOA1BP
ADAR	APOO
ADARB1	APRT
ADAT1	ARF5
ADCY6	ARL2
ADCY9	ARL6IP4
ADD1	ARMC6
ADD3	ARPC3
ADIPOR1	ARRDC1
ADIPOR2	ASPSCR1
ADNP	ATAD3B
ADPGK	ATG4D
ADSS	ATOX1
AEBP2	ATP5D
AFAP1	ATP5E
AFF1	ATP5G1

AFF4	ATP5G2
AFTPH	ATP5H
AGAP1	ATP5I
AGFG1	ATP5J2
AGGF1	ATP5O
AGL	ATP6V0C
AGPAT1	ATP6V1G1
AGPAT5	ATPIF1
AGPS	AUP1
AGRN	AURKAIP1
AHCTF1	AXIN2
AHCYL1	B3GAT3
AHR	B4GALT7
AIDA	B9D2
AIM1	BAD
AKAP10	BANF1
AKAP11	BAX
AKAP13	BCAP31
AKIRIN1	BCAT2
AKT1	BCL2L12
AKT3	BCL7C
ALAD	BCS1L
ALCAM	BGLAP
ALDH5A1	BID
ALDH9A1	BLOC1S1
ALG9	BLVRB
ALKBH5	BOLA3
AMD1	BRMS1
AMOT	BSCL2
AMOTL1	BSG
ANAPC1	BUD31
ANK3	C10orf11
ANKFY1	C11orf1
ANKIB1	C11orf10
ANKLE2	C11orf31
ANKMY2	C11orf48
ANKRD12	C11orf51
ANKRD13A	C11orf59
ANKRD13C	C11orf67
ANKRD17	C11orf73
ANKRD27	C11orf74
ANKRD28	C11orf83
ANKRD40	C12orf10
ANKRD46	C12orf45
ANKRD52	C12orf57
ANKRD6	C12orf62
ANO6	C13orf27
ANP32E	C14orf153

ANTXR2	C14orf156
AP1AR	C14orf179
AP1B1	C14orf2
AP1G1	C15orf63
AP2A2	C16orf13
AP2B1	C16orf42
AP3B1	C16orf48
AP3D1	C16orf53
AP3M1	C16orf61
AP3M2	C16orf68
APBB2	C17orf106
APH1A	C17orf37
API5	C17orf49
APOL6	C17orf61
APP	C17orf70
APPBP2	C17orf79
APPL1	C17orf81
APPL2	C17orf89
AQR	C18orf21
ARCN1	C19orf10
ARFGAP2	C19orf24
ARFGAP3	C19orf33
ARFGEF1	C19orf43
ARFGEF2	C19orf48
ARFIP1	C19orf53
ARHGAP12	C19orf56
ARHGAP17	C19orf60
ARHGAP21	C19orf66
ARHGAP24	C19orf70
ARHGAP26	C1orf151
ARHGAP29	C1orf31
ARHGAP31	C1orf35
ARHGAP42	C1orf54
ARHGAP5	C1orf66
ARHGEF10	C1orf86
ARHGEF12	C21orf57
ARHGEF18	C22orf32
ARHGEF3	C2orf28
ARHGEF6	C2orf7
ARHGEF7	C2orf76
ARID1A	C2orf79
ARID1B	C3orf75
ARID2	C4orf27
ARID4A	C4orf47
ARID4B	C6orf1
ARID5B	C6orf108
ARIH1	C6orf125
ARL5A	C6orf129

ARMC1	C6orf136
ARMC8	C6orf203
ARMCX3	C6orf26
ARNT	C7orf11
ARPP19	C7orf47
ARRDC3	C7orf50
ARRDC4	C7orf55
ARSD	C7orf59
ASAP1	C8orf30A
ASAP2	C8orf38
ASB8	C8orf40
ASCC1	C8orf41
ASCC3	C8orf59
ASH1L	C9orf100
ASH2L	C9orf116
ASPH	C9orf119
ASXL1	C9orf142
ATAD1	C9orf3
ATE1	C9orf89
ATF1	CAMTA1
ATF2	CAPG
ATF6	CAPN12
ATF7	CARD16
ATF7IP	CBR1
ATG9A	CCDC101
ATL3	CCDC106
ATMIN	CCDC107
ATP10D	CCDC12
ATP11A	CCDC124
ATP11B	CCDC142
ATP11C	CCDC159
ATP13A3	CCDC23
ATP2A2	CCDC24
ATP2B1	CCDC53
ATP2B4	CCDC56
ATP2C1	CCDC57
ATP6V0A1	CCDC58
ATP6V1B2	CCDC59
ATP6V1C1	CCDC61
ATP8A1	CCDC72
ATP9A	CCDC84
ATP9B	CCDC86
ATRN	CCDC94
ATRX	CCS
ATXN1L	CD320
ATXN7L3	CD63
AVL9	CDC34
AXL	CDK2AP2

AZIN1	CDK5
B3GALNT1	CENPT
B4GALT1	CEP290
B4GALT5	CHCHD1
BACE1	CHCHD5
BACH1	CHCHD8
BAHD1	CHEK2
BAT2	CHKB
BAT2L1	CHMP2A
BAT2L2	CHRNE
BAT3	CIB1
BAZ1B	CINP
BAZ2A	CISD3
BAZ2B	CKS2
BBS1	CLPP
BBX	CLTA
BCAS3	CLTB
BCL2L11	CNPY2
BCL7A	COASY
BCL9L	COMMD1
BCLAF1	COMMD4
BCOR	COMMD6
BDP1	COMTD1
BECN1	COPE
BEND7	COPS5
BHLHE41	COPS6
BICC1	COQ4
BICD1	CORO1B
BICD2	COX17
BIRC6	COX4I1
BIVM	COX5A
BLZF1	COX5B
BMP2K	COX6A1
BMPR2	COX6B1
BMS1	COX6C
BNIP2	COX7C
BPNT1	COX8A
BPTF	CPSF3L
BRAF	CPT1B
BRCC3	CREB3
BRD2	CRLS1
BRD3	CROCC
BRPF3	CSNK2B
BSDC1	CST3
BTBD1	CTDP1
BTBD10	CTU2
BTBD3	CUL9
BZW1	CUTA

C10orf119	CWC15
C10orf26	CYB561D2
C10orf46	CYBA
C10orf72	DAD1
C10orf76	DBI
C11orf54	DCI
C12orf23	DCXR
C13orf23	DDAH2
C14orf21	DDT
C14orf43	DDX39
C16orf62	DDX49
C16orf70	DDX56
C16orf72	DGCR6
C17orf85	DGCR6L
C18orf19	DGUOK
C19orf2	DHPS
C1orf106	DHRS7B
C1orf107	DHX34
C1orf144	DHX58
C1orf25	DMAP1
C1orf27	DMPK
C1orf55	DNAJC17
C1orf58	DNAJC4
C1orf9	DNLZ
C1QTNF9	DNTTIP1
C20orf194	DPM2
C22orf13	DPM3
C3orf17	DPP7
C3orf63	DPY30
C3orf64	DRAP1
C4orf34	DRG2
C4orf41	DTNB
C5orf22	DTX2
C5orf33	DTYMK
C5orf4	DUS1L
C5orf41	DUS3L
C6orf106	DUSP23
C6orf192	DYNLL1
C6orf211	DYNLRB1
C6orf62	DYNLT1
C6orf89	DYRK4
C7orf42	E4F1
C8orf83	EBP
C9orf129	ECH1
C9orf25	EDF1
C9orf5	EEF1D
C9orf64	EFHD2
CAB39	EGFL8

CACNA2D1	EIF3F
CADPS2	EIF3G
CALCRL	EIF3I
CALD1	EIF3K
CAMK2D	EIF4EBP1
CAMK2G	EIF4EBP3
CAMKK2	EIF5A
CAMSAP1	EIF6
CAMSAP1L1	ELOF1
CAND1	EMG1
CANX	EMP3
CAP1	ENDOG
CAP2	EPOR
CAPN2	ERCC1
CAPN7	ERH
CAPRIN1	ETFB
CAPZA1	ETHE1
CASC3	EXOSC1
CASC4	EXOSC4
CASD1	EXOSC5
CASP10	EXOSC7
CASP2	F8A1
CASP7	FADS3
CASP8	FAHD2A
CAST	FAM100A
CBFB	FAM100B
CBLL1	FAM108A1
CBX6	FAM113A
CCDC132	FAM128A
CCDC25	FAM128B
CCDC43	FAM158A
CCDC47	FAM162A
CCDC6	FAM165B
CCDC69	FAM173A
CCDC93	FAM176B
CCND1	FAM195A
CCNG2	FAM195B
CCNT1	FAM3A
CCNT2	FAM50A
CCNY	FAM58A
CCNYL1	FAM96B
CCPG1	FAM98C
CD109	FANCA
CD164	FASTK
CD2AP	FAU
CD302	FBF1
CD4	FBXL15
CD46	FBXL6

CD84	FBXW5
CD99L2	FDX1L
CDC14B	FER1L4
CDC23	FIBP
CDC27	FKBP2
CDC40	FKBP8
CDC42	FLAD1
CDC42BPA	FLJ35220
CDC42BPB	FLYWCH2
CDC42SE1	FUT5
CDC42SE2	G6PC3
CDC5L	GABARAP
CDC73	GADD45GIP1
CDH2	GALK1
CDH6	GALT
CDK12	GAMT
CDK13	GCAT
CDK14	GEMIN6
CDK17	GEMIN7
CDK19	GGTLC2
CDKAL1	GIYD2
CECR1	GLRX
CELF1	GLRX2
CELSR1	GLTSCR2
CEP120	GMPPA
CEP57	GNAS
CEP68	GNB2L1
CEPT1	GNG5
CERK	GNPTG
CHD1	GPAT2
CHD2	GPATCH1
CHD4	GPR137
CHD8	GPR172A
CHD9	GPS2
CHM	GPX1
CHMP7	GPX4
CHST9	GSDMD
CHTF8	GSTK1
CHUK	GSTO1
CKAP5	GTF2E2
CLASP1	GTF2H5
CLASP2	GTF3A
CLCC1	GTF3C6
CLCN3	GUK1
CLCN5	GZMA
CLDN12	GZMH
CLIC4	HAGH
CLINT1	HAUS1

CLIP1	HAX1
CLOCK	HBXIP
CLPX	HCFC1R1
CLSTN1	HCST
CLTC	HDDC3
CMIP	HDHD3
CMPK1	HEATR7A
CNBP	HEBP2
CNKSR3	HEMK1
CNN3	HES4
CNNM3	HEXIM2
CNOT1	HHLA3
CNOT2	HIGD2A
CNOT4	HINT2
CNOT6	HLA-F
CNOT6L	HMBS
CNOT7	HMG20B
CNOT8	HMOX2
CNST	HN1
COG2	HOMER3
COG3	HRAS
COG5	HSBP1
COG6	HSCB
COL4A3BP	HSD17B10
COPA	HSPB1
COPB1	HSPB11
COPS2	HSPBP1
CORO1C	HSPE1
COX15	HYI
CPA3	ICAM3
CPD	ICT1
CPEB2	IDUA
CPEB4	IFI27
CPNE3	IFI27L1
CPNE8	IFI27L2
CPSF2	IFI35
CPSF6	IFITM3
CPT1A	IFRD2
CPT2	IFT20
CRCP	IFT27
CREB1	IL18BP
CREB3L2	IL32
CREBBP	ILKAP
CREBL2	IMMP1L
CREG1	INPP5E
CRIM1	IRF3
CRK	IRF7
CRKL	ISG15

CRLF3	ISG20
CRNKL1	ISOC2
CRTC3	ISYNA1
CS	ITGAE
CSDE1	ITGB3BP
CSE1L	ITPA
CSF1R	JOSD2
CSGALNACT2	JTB
CSNK1A1	KAT2A
CSNK1G3	KCTD13
CSNK2A1	KIAA1731
CTBP2	KIF12
CTBS	KIFC2
CTCF	KNTC1
CTDSP2	KRT18
CTDSPL2	KRT8
CTNNA1	KRTCAP2
CTNNB1	LENG1
CTNND1	LGALS1
CTR9	LIMD2
CTSO	LIME1
CTSS	LIN37
CTTN	LOC100288778
CTTNBP2NL	LOC150381
CUBN	LOC150776
CUL2	LOC375190
CUL3	LOC388789
CUL4A	LOC388796
CUL4B	LOC440957
CUL5	LOC550643
CWC22	LOC728855
CWF19L2	LOC728875
CXorf38	LRRC23
CXorf56	LRRC45
CYB5D1	LRWD1
CYB5R1	LSM1
CYBASC3	LSM10
CYBB	LSM3
CYFIP1	LSM4
CYLD	LSM7
CYP4V2	LSMD1
CYP7B1	LST1
CYTH1	LTB
CYTH3	LUC7L
CYTSA	LZTS2
DAB2	MACROD1
DAPK1	MAD2L2
DARS	MAF1

DARS2	MAGOH
DAZAP2	MAGOHB
DBT	MANF
DCAF10	MAP2K2
DCAF12	MAPK7
DCAF5	2-Mar
DCAF6	MCTS1
DCAF7	MDP1
DCAF8	ME3
DCTD	MEA1
DCTN1	MED11
DCTN4	MED25
DCTN5	MED27
DCUN1D1	METRN
DCUN1D4	METTL1
DDAH1	METTL11A
DDB1	METTL3
DDHD2	METTL5
DDX1	MFSD3
DDX17	MGMT
DDX18	MGST2
DDX19A	MGST3
DDX21	MIA
DDX23	MICALL2
DDX3X	MIF
DDX42	MIIP
DDX46	MITD1
DDX50	MLST8
DDX58	MORN2
DDX6	MPG
DDX60	MPV17
DEK	MPV17L2
DENND1A	MRPL11
DENND4C	MRPL12
DENND5A	MRPL14
DENR	MRPL16
DGCR2	MRPL17
DHTKD1	MRPL18
DHX15	MRPL2
DHX36	MRPL20
DHX38	MRPL21
DHX40	MRPL22
DHX8	MRPL23
DHX9	MRPL24
DIAPH1	MRPL27
DICER1	MRPL28
DIDO1	MRPL36
DIP2B	MRPL38

DIP2C	MRPL40
DIS3	MRPL41
DIS3L	MRPL47
DIXDC1	MRPL48
DLAT	MRPL51
DLC1	MRPL52
DLG1	MRPL53
DMD	MRPL54
DMXL1	MRPL55
DNAJA2	MRPS11
DNAJB14	MRPS15
DNAJC10	MRPS21
DNAJC11	MRPS24
DNAJC13	MRPS26
DNAJC16	MRPS34
DNAJC3	MRPS36
DNAJC5	MSH5
DNM1L	MSRB2
DOCK1	MTCP1NB
DOCK4	MTERFD1
DOCK7	MTX1
DOCK8	MUTYH
DOCK9	MXD3
DPP4	MYEOV2
DPP8	MYL12B
DPY19L1	MYL5
DPY19L4	MYL6
DPYD	MYL6B
DPYSL2	N6AMT2
DSC2	NAA10
DSCR3	NANS
DSP	NAPRT1
DST	NARFL
DTX3L	NCAPH2
DUSP16	NCRNA00116
DUSP3	NCRNA00152
DYM	NDUFA1
DYNC1H1	NDUFA11
DYNC1I2	NDUFA12
DYNC1LI2	NDUFA13
DYNLL2	NDUFA2
DYRK1A	NDUFA3
DYSF	NDUFA7
DYX1C1	NDUFA8
EAF1	NDUFAF2
EARS2	NDUFAF3
EDEM1	NDUFB1
EDEM3	NDUFB10

EDIL3	NDUFB11
EEA1	NDUFB2
EFNB2	NDUFB4
EFR3A	NDUFB6
EFTUD1	NDUFB7
EFTUD2	NDUFB8
EGFR	NDUFB9
EGLN1	NDUFC1
EHBP1	NDUFC2
EHHADH	NDUFS5
EHMT1	NDUFS6
EI24	NDUFS7
EIF2AK1	NDUFS8
EIF2AK2	NDUFV2
EIF2AK3	NEDD8
EIF2AK4	NENF
EIF2C1	NEURL4
EIF2S1	NFKBIB
EIF2S3	NHP2
EIF3A	NHP2L1
EIF3J	NIT2
EIF4B	NME1-NME2
EIF4E3	NME1
EIF4EBP2	NME2
EIF4ENIF1	NME3
EIF4G1	NME4
EIF4G2	NMRAL1
EIF4G3	NOC4L
EIF4H	NOL12
EIF5	NOP10
ELAVL1	NOP16
ELF1	NOSIP
ELF2	NOXA1
ELF4	NPR2
ELK3	NPRL2
ELMOD2	NR2C2AP
ELOVL5	NR2F6
ELP2	NSUN5
EML1	NSUN5P1
EML4	NSUN5P2
ENAH	NT5C
ENPEP	NUBP2
ENPP4	NUDC
EP300	NUDT1
EP400	NUDT14
EPAS1	NUDT2
EPB41L2	NUDT22
EPB41L4A	NUDT5

EPC1	NUDT8
EPC2	NUPR1
EPHA7	NUTF2
EPN2	OAF
EPRS	OAZ1
EPS15	OCEL1
ERAP1	ODF3B
ERBB2IP	ORMDL2
ERC1	OSGEP
ERGIC1	OST4
ERLIN1	OTUB1
ERLIN2	P2RX5
ERMP1	P4HTM
ERO1LB	PABPC1L
ESYT1	PAFAH1B3
ESYT2	PARK7
ETF1	PCBP4
ETNK1	PCGF1
ETS1	PCNT
ETV5	PCYT2
EVC	PDCD5
EXD2	PDE6D
EXOC1	PDRG1
EXOC2	PDZD11
EXOC4	PEX16
EXOC5	PFDN2
EXOC6	PFDN4
EXOC6B	PFDN5
EZH1	PFDN6
F11R	PFN1
FAF1	PGLS
FAF2	PHKG2
FAM107B	PHLDA3
FAM114A2	PHPT1
FAM115A	PIH1D1
FAM116A	PIN1
FAM120A	PIN4
FAM120B	PIR
FAM122B	PKMYT1
FAM129A	PL-5283
FAM129B	PLA2G16
FAM134C	PLEKHO1
FAM13A	PLP2
FAM13B	PMF1
FAM149B1	PNKP
FAM160B1	POLD1
FAM168A	POLD4
FAM168B	POLE4

FAM171A1	POLG2
FAM172A	POLR2F
FAM175B	POLR2G
FAM178A	POLR2H
FAM18B	POLR2I
FAM190B	POLR2J
FAM198B	POLR2L
FAM199X	POLRMT
FAM20B	POMP
FAM21C	POP5
FAM35A	POP7
FAM38A	PPAN-P2RY11
FAM53C	PPAN
FAM59A	PPDPF
FAM73A	PPIA
FAM8A1	PPIB
FAM91A1	PPIH
FAM98A	PPOX
FAM98B	PPP1CA
FANCC	PPP1R14B
FAR1	PPP1R16A
FAS	PPP4C
FAT1	PPPDE2
FBXL17	PQBP1
FBXL3	PQLC2
FBXL4	PRDX1
FBXL5	PRDX5
FBXO11	PRELID1
FBXO21	PRICKLE4
FBXO28	PRKCDBP
FBXO3	PRMT1
FBXO38	PRR5
FBXW11	PSENEN
FBXW2	PSMA3
FCF1	PSMA4
FCHO2	PSMA7
FCHSD2	PSMB1
FECH	PSMB10
FERMT2	PSMB3
FEZ2	PSMB4
FGD4	PSMB5
FGFRL1	PSMB6
FGL2	PSMB8
FKBP15	PSMB9
FKBP9	PSMC3
FLI1	PSMC3IP
FLNA	PSMC4
FLOT2	PSMC5

FLT1	PSMD13
FMNL2	PSMD6
FMR1	PSMD8
FNBP1	PSMD9
FNBP1L	PSME1
FNDC3A	PSME2
FNDC3B	PSMG4
FNIP1	PTCD1
FNIP2	PTGES2
FOXJ2	PTOV1
FOXJ3	PTPRCAP
FOXN3	PTRH1
FPGT	PTTG1
FPR3	PUS1
FRMD3	PYCARD
FRMD4A	QTRT1
FRMD4B	RAB24
FRYL	RAB4B
FSTL1	RABAC1
FTO	RABEP2
FTSJD2	RABGGTA
FUBP1	RAD9A
FUBP3	RAG1AP1
FUCA1	RALY
FURIN	RANGRF
FXR2	RARRES2
FYCO1	RARRES3
FYTTD1	RASSF7
G3BP1	RBM42
G3BP2	RBPMS
GAB1	RBX1
GAB2	RDBP
GABPA	RDH5
GALC	REXO1
GALNT1	RFNG
GALNT10	RFXANK
GANAB	RGS14
GAPVD1	RHOC
GART	RHOD
GAS2L3	RHPN1
GATAD2A	RILP
GBE1	RNASEH2C
GBF1	RNASEK
GCC2	RNASET2
GCLC	RNF181
GCLM	RNF25
GCN1L1	RNF7
GDI2	ROBLD3

GEMIN5	ROGDI
GFM1	ROMO1
GFPT1	RP9
GGA2	RPAP1
GGCX	RPL11
GGNBP2	RPL12
GIGYF2	RPL13
GIT2	RPL13A
GLE1	RPL14
GLG1	RPL17
GLUL	RPL18
GLYR1	RPL18A
GM2A	RPL19
GMCL1	RPL23
GMFB	RPL23A
GNA12	RPL24
GNA13	RPL26L1
GNAI1	RPL27
GNAI3	RPL27A
GNAQ	RPL28
GNB1	RPL29
GNB4	RPL3
GNE	RPL30
GNG12	RPL32
GNPDA2	RPL35
GNPTAB	RPL35A
GNS	RPL36
GOLGA2	RPL36A
GOLGA3	RPL37
GOLIM4	RPL37A
GOLPH3	RPL38
GOLPH3L	RPL39
GOPC	RPL5
GORASP2	RPL6
GOSR1	RPL7A
GPBP1	RPL8
GPBP1L1	RPL9
GPC6	RPLP1
GPD2	RPLP2
GPR107	RPP21
GPR125	RPP30
GPR126	RPS10
GPR155	RPS11
GPR89A	RPS13
GRAMD3	RPS14
GRB2	RPS15
GRLF1	RPS16
GRSF1	RPS17

GSK3B	RPS19	
GSPT1	RPS19BP1	
GTF2A1	RPS2	
GTF2H3	RPS20	
GTF2I	RPS21	
GTF3C1	RPS23	
GTF3C2	RPS25	
GTF3C3	RPS26	
GTPBP1	RPS27L	
GUCY1A3	RPS3	
GUF1	RPS6	
H6PD	RPS6KB2	
HADHA	RPS7	
HAT1	RPS8	
HBS1L	RPS9	
HCFC1	RPSA	
HCFC2	RPUSD3	
HDAC2	RRAS	
HDHD2	RRP7A	
HDLBP	RRP7B	
HEATR5B	RRP8	
HECTD1	RRP9	
HECW2	RTEL1	
HEG1	RTN4IP1	
HELZ	RUVBL2	
HERC1	RWDD3	
HERC3	S100A10	
HERC4	S100A11	
HERPUD2	S100A13	
HFE	S100A6	
HGSNAT	SARNP	
HIAT1	SAT2	
HIATL1	SCNM1	
HIF1A	SCO2	
HIF1AN	SCRN2	
HINT3	SDF2L1	
HIP1	SDR39U1	
HIPK1	SEC13	
HIPK2	SEC31B	
HIPK3	SEC61B	
HIRA	SELK	
HIVEP2	SELM	
HK1	SELO	
HK2	1-Sep	
HLTF	SEPW1	
HMG20A	SEPX1	
HMGCR	SERF2	
HMGCS1	SF3A2	

HMGXB3	SFI1
HMGXB4	SFRS12IP1
HN1L	SFRS16
HNRNPF	SFRS8
HNRNPK	SH3BGRL3
HNRNPM	SHARPIN
HNRNPR	SHFM1
HNRNPU	SIGIRR
HNRNPUL1	SIRT6
HOOK1	SIRT7
HP1BP3	SIVA1
HS2ST1	SLC22A18
HSD17B4	SLC25A1
HSP90AA1	SLC25A39
HSP90AB1	SLC2A4RG
HSPA12A	SLC39A4
HSPA13	SMPD2
HSPA4L	SNAPC2
HSPC159	SNHG6
HTATSF1	SNHG9
HTT	SNORA8
HUWE1	SNRNP25
HYOU1	SNRNP35
IARS	SNRPA
IARS2	SNRPB
IBTK	SNRPB2
ICMT	SNRPD2
IDE	SNRPF
IDS	SNRPG
IFNAR1	SOD1
IFT88	SPA17
IGF1R	SPAG4
IGF2R	SPSB3
IKBKAP	SRA1
IL13RA1	SRM
IL17RA	SRP14
IL1R1	SS18L2
IL4R	SSBP1
IL6R	SSBP4
IL6ST	SSNA1
ILF3	SSR4
IMMT	SSSCA1
IMPACT	SSU72
INADL	STARD10
ING3	STOML1
INO80	STOML2
INPP5A	STUB1
INSR	STX10

INTS6	STX8
IP6K1	STYXL1
IPO11	SULT1A1
IPO5	SULT1A3
IPO7	SURF1
IPO8	SURF2
IPO9	SYCE1L
IQGAP1	TACC3
IQGAP2	TAF10
IQSEC1	TALDO1
IREB2	TARBP1
ITCH	TARBP2
ITFG1	TAX1BP3
ITGA1	TAZ
ITGA2	TBC1D10C
ITGA4	TBC1D24
ITGA6	TBC1D3B
ITGA8	TBC1D3C
ITGAV	TBC1D3G
ITGB1	TBC1D3H
ITGB8	TBCA
ITSN1	TBCB
ITSN2	TBRG4
JAG1	TCEA2
JAK1	TCEB1
JAZF1	TCEB2
JKAMP	TCTEX1D2
JMJD1C	TECR
JOSD1	TELO2
JUB	TEX264
KAT2B	TFPT
KAZ	THAP4
KBTBD2	THAP7
KCNIP4	THOC6
KCNJ16	THOC7
KCNJ3	THOP1
KCNMA1	THYN1
KCTD10	TIMM10
KCTD18	TIMM13
KCTD2	TIMM16
KCTD20	TIMM17B
KCTD3	TIMM44
KCTD5	TIMM50
KCTD9	TM4SF5
KDELC2	TM7SF2
KDM1B	TMED1
KDM2A	TMED3
KDM3A	TMEM115

KDM3B	TMEM134
KDM4A	TMEM141
KDM4C	TMEM147
KDM5A	TMEM149
KDM5B	TMEM176A
KDM5C	TMEM179B
KDM6A	TMEM191A
KDR	TMEM205
KDSR	TMEM208
KHDRBS1	TMEM219
KHSRP	TMEM223
KIAA0040	TMEM39B
KIAA0090	TMEM54
KIAA0100	TMEM60
KIAA0146	TMSB10
KIAA0174	TMUB1
KIAA0196	TNFRSF6B
KIAA0232	TOMM40
KIAA0247	TOMM5
KIAA0317	TOMM6
KIAA0319L	TP53I13
KIAA0355	TP53I3
KIAA0368	TP53TG1
KIAA0427	TPRA1
KIAA0430	TPRN
KIAA0494	TRAPPC1
KIAA0528	TRAPPC2L
KIAA0562	TRAPPC2P1
KIAA0652	TRAPPC4
KIAA0776	TRAPPC6A
KIAA0892	TRMT1
KIAA1012	TRMT112
KIAA1033	TRMT2A
KIAA1109	TRMU
KIAA1147	TRPM4
KIAA1191	TRPT1
KIAA1217	TSEN54
KIAA1267	TSPO
KIAA1279	TSSC4
KIAA1370	TST
KIAA1429	TSTA3
KIAA1430	TSTD1
KIAA1462	TTLL1
KIAA1598	TUSC2
KIAA1671	TXN
KIAA1715	TXNDC17
KIDINS220	TXNL4A
KIF13A	U2AF1L4

KIF16B	UBA52
KIF1B	UBE2M
KIF3B	UBE2S
KIF5B	UBL5
KIFAP3	UBL7
KIRREL	UBXN1
KITLG	UFD1L
KL	ULK3
KLF11	UQCR10
KLF3	UQCR11
KLF6	UQCRB
KLHDC10	UQCRC1
KLHL12	UQCRQ
KLHL2	UROD
KLHL20	USE1
KLHL24	UXT
KLHL5	VAMP5
KLHL7	VAMP8
KLRAQ1	VEGFB
KPNA1	VPS28
KPNA3	WASH2P
KPNA4	WASH3P
KPNA6	WASH5P
KPNB1	WASH7P
KRAS	WBSCR22
KRR1	WDR13
KSR1	WDR18
L3MBTL2	WDR24
LACTB	WDR34
LAMC1	WDR45
LAMP1	WDR74
LAMP2	WDR83
LANCL1	WDR90
LAPTM5	WIBG
LARP1	WRAP53
LARP1B	XPNPEP3
LARP4	YDJC
LARP4B	YIF1A
LARS	YIF1B
LASP1	YIPF2
LASS2	YPEL3
LASS6	ZBTB17
LATS2	ZBTB48
LBR	ZBTB8OS
LCP1	ZC3H3
LEMD3	ZDHHC12
LEPR	ZDHHC24
LEPROT	ZFAND2A

LGALS8
LGR4
LIFR
LIMCH1
LIMD1
LIN7C
LIPA
LIX1L
LMAN1
LMAN2L
LMBR1
LMBRD2
LNPEP
LOC339524
LOC651250
LOC729082
LONP2
LPGAT1
LPHN2
LPIN2
LRBA
LRCH1
LRCH3
LRIG1
LRIG3
LRP1
LRP10
LRP2
LRPPRC
LRRC40
LRRC58
LRRK2
LSG1
LSM14A
LTA4H
LUZP6
LYN
LYSMD3
M6PR
MACF1
MADD
MAGT1
MAK16
MALT1
MAML1
MAML2
MAN1A1
MAN1A2
ZFPL1
ZMAT5
ZMYND19
ZNF32
ZNF335
ZNF414
ZNF428
ZNF444
ZNF511
ZNF524
ZNF585A
ZNF593
ZNF688
ZNF692
ZNF706
ZNF787
ZNF8
ZNHIT1
ZNRD1
ZP3

MAN2A1
MAN2B2
MANBA
MANEA
MAP2K4
MAP3K1
MAP3K2
MAP3K3
MAP3K5
MAP3K7
MAP4
MAP4K4
MAP4K5
MAPK1
MAPK14
MAPK1IP1L
MAPK6
MAPK8
MAPK9
MAPRE1
MAPRE2
1-Mar
6-Mar
7-Mar
8-Mar
MARK2
MARK4
MASP1
MAT2A
MAT2B
MATR3
MAVS
MBD1
MBNL1
MBNL2
MBP
MBTPS1
MBTPS2
MCCC1
MCCC2
MCFD2
MCM3
MCM3AP
MDFIC
MDM2
ME2
MED1
MED13

MED13L
MED14
MED17
MED23
MED26
MEF2A
MEF2C
MEF2D
MEGF8
MERTK
MET
METAP1
METAP2
METTL13
METTL14
MFAP1
MFAP3
MFN1
MFN2
MFSD1
MFSD11
MFSD6
MGAT4A
MGAT5
MGEA5
MGLL
MGRN1
MIA2
MIB1
MICAL2
MICAL3
MID1
MIER1
MINPP1
MITF
MKLN1
MKRN1
MLL
MLL3
MLL5
MLLT10
MLLT4
MLXIP
MMGT1
MOBKL1B
MOBKL2B
MON1B
MON2

MORC3
MPDZ
MPP5
MSH2
MSI2
MSN
MTAP
MTDH
MTFR1
MTIF2
MTMR1
MTMR10
MTMR12
MTMR2
MTMR3
MTMR6
MTO1
MTOR
MTR
MTRF1L
MTRR
MTSS1
MUT
MUTED
MXI1
MYCBP2
MYLIP
MYLK
MYO18A
MYO1C
MYO1D
MYO1E
MYO5A
MYO6
MYO9A
MYOF
MYST2
N4BP1
NAA15
NAA30
NAA35
NAA50
NAB1
NAF1
NARS
NAT10
NBAS
NBN

NBPF1
NBR1
NCEH1
NCK2
NCKAP1
NCOA1
NCOA2
NCOA3
NCOA4
NCOA5
NCOA7
NCOR1
NCOR2
NCSTN
NDFIP2
NDRG1
NDRG3
NDST1
NEBL
NECAP1
NECAP2
NEDD1
NEDD4
NEDD9
NEK7
NEO1
NETO2
NF1
NFATC3
NFE2L1
NFE2L2
NFIA
NFIB
NFIX
NFKB1
NFX1
NFYA
NGLY1
NHLRC3
NID1
NIPA2
NIPAL2
NIPAL3
NIPBL
NLK
NLN
NMD3
NMT2

NOL10
NOL11
NOLC1
NOMO1
NOMO2
NOMO3
NONO
NOP14
NOTCH2
NPC1
NPEPPS
NPLOC4
NR1D2
NR3C1
NRAS
NRD1
NRP1
NSD1
NSF
NSL1
NSMAF
NSUN2
NT5C2
NUAK1
NUB1
NUDCD3
NUFIP2
NUMB
NUP133
NUP153
NUP155
NUP160
NUP205
NUP214
NUP43
NUP50
NUP54
NUP98
NUPL1
NUS1
OAS3
OCRL
OGDH
OLFML2A
OPA1
ORAI2
ORC2L
OS9

OSBP
OSBPL10
OSBPL1A
OSBPL8
OSBPL9
OSGIN2
OSMR
OTUD4
OTUD7B
OXR1
OXSR1
P4HA1
PACSIN2
PAFAH1B1
PAFAH1B2
PAFAH2
PAG1
PAICS
PAK1
PAK2
PAM
PAN3
PANK1
PANK3
PAPD4
PAPOLA
PAPSS1
PAPSS2
PARD3
PARG
PARK2
PARN
PARP1
PARP14
PARP4
PARP8
PATL1
PBLD
PBX1
PCDHGB7
PCDHGC3
PCGF5
PCM1
PCMTD1
PCMTD2
PCNX
PCYOX1
PCYT1A

PDCD6IP
PDCD7
PDCL
PDE4A
PDE4B
PDE4D
PDE7A
PDE8A
PDGFC
PDK1
PDLIM5
PDPK1
PDS5A
PDS5B
PDSS2
PDXDC1
PDZD8
PELI1
PER3
PEX11B
PEX26
PEX5
PGGT1B
PGM2
PGM3
PGRMC2
PHACTR2
PHACTR4
PHAX
PHF10
PHF15
PHF17
PHF2
PHF20
PHF20L1
PHF21A
PHF3
PHF8
PHIP
PHKB
PHLDB2
PHRF1
PHTF1
PI4K2A
PI4K2B
PI4KA
PIAS1
PICALM

PIGG
PIGK
PIGN
PIGS
PIGX
PIK3AP1
PIK3C2A
PIK3C3
PIK3CA
PIK3CB
PIK3R1
PIK3R3
PIKFYVE
PIP4K2A
PIP4K2B
PIP4K2C
PIP5K1A
PIP5K1C
PITPNA
PITPNB
PITRM1
PJA2
PKD2
PKN2
PKP2
PKP4
PLAA
PLBD2
PLCB1
PLD1
PLDN
PLEC
PLEKHA1
PLEKHA2
PLEKHA3
PLEKHA7
PLEKHG1
PLEKHO2
PLS1
PLSCR4
PLXDC2
PLXNA2
PLXNC1
PM20D2
PMS2
PNPLA3
PNRC2
POC1B

PODXL
POFUT1
POGK
POLD3
POLDIP3
POLH
POLK
POLR2A
POLR2B
POM121
POM121C
PPAP2B
PPARD
PPFIA1
PPFIBP1
PPIG
PPIP5K2
PPM1B
PPM1D
PPM1F
PPME1
PPP1CB
PPP1CC
PPP1R10
PPP1R12A
PPP1R16B
PPP1R8
PPP2CB
PPP2R1B
PPP2R2A
PPP2R3A
PPP2R5D
PPP2R5E
PPP3CA
PPP3R1
PPP4R1
PPP4R2
PPP6C
PPT1
PPTC7
PRCP
PRDM4
PREPL
PREX1
PRKAA1
PRKAA2
PRKACB
PRKAR1A

PRKAR2A
PRKCI
PRKD1
PRKD3
PRKDC
PRKX
PRMT5
PROSC
PRPF18
PRPF4
PRPF40A
PRPF4B
PRPF8
PRRC1
PRUNE
PRUNE2
PSAP
PSEN1
PSIP1
PSMD1
PSMD12
PSMD5
PSME4
PTAR1
PTBP1
PTDSS1
PTEN
PTK2
PTP4A1
PTPLB
PTPN1
PTPN11
PTPN12
PTPN13
PTPN14
PTPN23
PTPN9
PTPRB
PTPRE
PTPRG
PTPRJ
PTPRK
PTPRM
PUM1
PUM2
PVRL3
PWP1
PXN

PYROXD1
QKI
QRICH1
R3HDM1
R3HDM2
RAB10
RAB12
RAB14
RAB18
RAB1A
RAB21
RAB31
RAB35
RAB36
RAB3GAP1
RAB3GAP2
RAB5A
RAB5B
RAB6A
RAB7L1
RAB8B
RABEP1
RABGAP1
RABGEF1
RABL3
RAD17
RAD21
RAD23B
RAD50
RAI14
RALB
RALBP1
RALGAPA2
RALGAPB
RANBP10
RANBP2
RANBP9
RAP1A
RAP1B
RAPGEF1
RAPGEF2
RAPGEF5
RARS
RASA1
RASA3
RASGRP1
RASSF2
RASSF3

RAVER2
RB1
RB1CC1
RBBP4
RBBP5
RBBP9
RBL2
RBM12
RBM16
RBM18
RBM22
RBM23
RBM26
RBM27
RBM47
RBM7
RBMS1
RBMS2
RBPJ
RC3H2
RCBTB1
RCBTB2
RCC2
RCHY1
RCOR1
RCOR3
RCSD1
RDX
RECQL
REEP3
RELL1
REPS1
RERE
RETSAT
RFC1
RFFL
RFX5
RGL1
RGNEF
RGP1
RHBDD1
RHOA
RHOBTB1
RHOBTB3
RHOT1
RHOU
RHPN2
RIF1

RIN2
RIOK2
RIOK3
RIPK1
RIT1
RMND5A
RNASEN
RNF103
RNF11
RNF111
RNF115
RNF121
RNF128
RNF13
RNF138
RNF141
RNF144B
RNF145
RNF146
RNF160
RNF170
RNF185
RNF19B
RNF20
RNF216
RNF38
RNF4
RNF40
RNF6
RNMT
ROCK1
ROCK2
ROD1
RORA
RP2
RPA1
RPE
RPL7L1
RPRD1A
RPRD1B
RPRD2
RPS6KA2
RPS6KA3
RPS6KB1
RPUSD4
RRM1
RRM2B
RRN3

RRP1B
RSBN1L
RSF1
RSPRY1
RTN3
RUNDC2A
RYBP
RYK
SAMD4B
SAMHD1
SAP130
SAPS3
SART3
SASH1
SAV1
SBF2
SBNO1
SCAMP1
SCAPER
SCARB2
SCD
SCP2
SCRN1
SCRN3
SCYL2
SDAD1
SDCBP
SDCCAG8
SEC14L1
SEC16A
SEC22B
SEC23A
SEC23B
SEC23IP
SEC24A
SEC24B
SEC24C
SEC24D
SEC31A
SEC61A1
SEC63
SECISBP2L
SEH1L
SEL1L
SEMA4D
SEMA5A
SEMA6A
SENP2

SENP6
SEPN1
SEPP1
SEPSECS
10-Sep
11-Sep
2-Sep
7-Sep
8-Sep
9-Sep
SERINC1
SERINC3
SERINC5
SERPINB9
SESTD1
SETD3
SETD7
SETX
SF1
SF3A1
SF3A3
SF3B3
SFRS13A
SFRS2IP
SFXN1
SFXN3
SGK3
SGMS1
SGMS2
SGPL1
SGPP2
SGSH
SH2B3
SH3BGRL2
SH3D19
SHOC2
SHROOM4
SIAE
SIK2
SIK3
SIN3A
SIPA1L2
SIRT1
SKAP2
SKI
SKIL
SKIV2L2
SLAIN2

SLC11A2
SLC12A2
SLC12A6
SLC12A7
SLC12A8
SLC15A4
SLC16A1
SLC16A4
SLC17A5
SLC1A1
SLC1A3
SLC20A2
SLC23A2
SLC25A13
SLC25A24
SLC25A30
SLC25A36
SLC25A44
SLC25A46
SLC30A5
SLC30A6
SLC30A7
SLC30A9
SLC33A1
SLC35A3
SLC35A5
SLC35B3
SLC35B4
SLC35D1
SLC35E1
SLC35F5
SLC37A2
SLC37A3
SLC38A1
SLC38A2
SLC39A10
SLC39A8
SLC39A9
SLC40A1
SLC41A1
SLC41A2
SLC4A4
SLC6A6
SLC8A1
SLCO2B1
SLCO4C1
SLFN5
SLK

SLMAP
SLU7
SMAD3
SMAD4
SMAD5
SMAP1
SMAP2
SMARCA1
SMARCA2
SMARCA5
SMARCAD1
SMARCC1
SMARCC2
SMARCD1
SMC1A
SMC3
SMCHD1
SMCR7L
SMEK1
SMEK2
SMG1
SMG6
SMG7
SMPD4
SMU1
SMURF1
SMURF2
SNAP23
SNAPC3
SNRK
SNRNP200
SNX1
SNX10
SNX12
SNX13
SNX19
SNX2
SNX25
SNX27
SNX29
SNX30
SNX4
SNX6
SNX9
SOAT1
SON
SORBS1
SORBS2

SORL1
SORT1
SOS1
SOS2
SP1
SP2
SP3
SPAG9
SPAST
SPATA13
SPATA18
SPATA6
SPATS2
SPDYA
SPG11
SPG20
SPIN1
SPIRE1
SPOP
SPOPL
SPPL2A
SPPL3
SPRED1
SPRED2
SPTAN1
SPTBN1
SPTLC1
SPTY2D1
SR140
SRBD1
SRCAP
SRFBP1
SRGAP2
SRP68
SRP72
SRP9
SRPK1
SRPK2
SRPR
SRRM1
SS18
SSFA2
SSR1
SSX2IP
ST13
ST3GAL1
ST8SIA4
STAG1

STAG2
STAM
STAM2
STARD13
STARD7
STARD8
STAT3
STAT5B
STAT6
STAU1
STAU2
STK10
STK17B
STK24
STK32B
STK38
STK38L
STK4
STK40
STOM
STRN
STRN3
STS
STT3A
STT3B
STX12
STX17
STX2
STX3
STX6
STX7
STXBP3
SUCLG2
SUDS3
SUFU
SUN1
SUN2
SUPT16H
SUPT6H
SUV420H1
SUZ12
SWAP70
SYK
SYNCRIP
SYNE1
SYNE2
SYNM
SYNRG

SYPL1
TAB1
TAB2
TAB3
TACC1
TAF1
TAF1B
TAF2
TAF9B
TANC1
TANK
TAOK1
TAOK3
TAPBP
TAPT1
TARDBP
TARSL2
TBC1D13
TBC1D15
TBC1D16
TBC1D19
TBC1D20
TBC1D22B
TBC1D23
TBC1D2B
TBC1D5
TBC1D8B
TBC1D9
TBC1D9B
TBCK
TBK1
TBL1X
TBL1XR1
TCEA1
TCF12
TCF7L2
TCHP
TCTN2
TCTN3
TDG
TDP2
TEAD1
TERF1
TERF2
TEX2
TEX261
TFCP2
TFDP1

TFRC
TGFA
TGFBR1
TGFBR2
TGFBR3
TGFBRAP1
TGOLN2
TGS1
THBS1
THOC2
THRAP3
THUMPD1
TIMM17A
TJP1
TJP2
TK2
TLE3
TLK1
TLK2
TLN1
TLN2
TLR3
TLR4
TM7SF3
TM9SF2
TM9SF3
TM9SF4
TMCC3
TMCO3
TMED2
TMED5
TMED7-
TICAM2
TMED7
TMEM123
TMEM127
TMEM131
TMEM135
TMEM144
TMEM150C
TMEM167B
TMEM181
TMEM184B
TMEM185A
TMEM192
TMEM2
TMEM200A
TMEM209

TMEM30A
TMEM41B
TMEM43
TMEM48
TMEM57
TMEM63B
TMEM66
TMEM87A
TMEM87B
TMF1
TMOD3
TMPO
TMTC1
TMTC2
TMTC3
TMX1
TMX3
TNFAIP1
TNFRSF10A
TNFRSF19
TNFRSF1B
TNKS
TNKS2
TNNI3K
TNPO1
TNPO2
TNPO3
TNS1
TNS3
TOM1L2
TOP1
TOP2B
TOR1AIP1
TOR1B
TOX4
TP53
TP53BP2
TPMT
TPP1
TPP2
TPR
TPRG1L
TRA2B
TRAF3IP1
TRAF6
TRAK2
TRAM1
TRAM2

TRAPPC10
TRAPPC6B
TRIM23
TRIM25
TRIM26
TRIM33
TRIM4
TRIM44
TRIM56
TRIO
TRIP12
TRPM7
TRRAP
TSN
TSNAX-DISC1
TSNAX
TSPAN12
TSPAN9
TSR1
TTC17
TTC19
TTC28
TTC3
TTC33
TTC37
TUBB
TUBGCP3
TUG1
TULP3
TXLNA
TXLNG
TXNDC5
TXNIP
TYW1
UBA6
UBE2D3
UBE2G1
UBE2G2
UBE2H
UBE2J1
UBE2W
UBE2Z
UBE3A
UBE3B
UBE3C
UBE4A
UBE4B
UBLCP1

UBN1
UBP1
UBQLN1
UBR1
UBR2
UBR3
UBR4
UBR5
UBR7
UBTD2
UBXN2B
UBXN4
UCHL5
UEVLD
UGCG
UGGT1
UGT2A3
UHMK1
ULK2
UNC119B
UNC13B
UNC5B
UNG
UPF1
UPF2
UPRT
USO1
USP1
USP10
USP12
USP14
USP15
USP16
USP19
USP22
USP24
USP25
USP3
USP30
USP32
USP33
USP34
USP36
USP38
USP40
USP47
USP48
USP53

USP7
USP8
USP9X
UTRN
UVRAG
VAMP3
VAMP7
VAPB
VAV2
VAV3
VCL
VEZF1
VEZT
VIPAR
VPS13C
VPS24
VPS26A
VPS35
VPS36
VPS39
VPS41
VPS4B
VPS52
VPS54
VRK2
VTA1
VWA5A
WAC
WAPAL
WARS2
WASF2
WASL
WBP11
WDFY1
WDFY3
WDR11
WDR26
WDR3
WDR33
WDR36
WDR43
WDR45L
WDR48
WDR55
WDR72
WDR82
WDTC1
WHSC1L1

WIPF1
WIPF2
WNK1
WRB
WWC2
WWC3
WWOX
WWP1
WWP2
WWTR1
XIAP
XPC
XPNPEP1
XPO1
XPO5
XPO6
XPO7
XPR1
XRCC5
XRCC6
XRN1
XRN2
YAP1
YEATS2
YES1
YIPF5
YIPF6
YLPM1
YME1L1
YPEL2
YTHDC1
YTHDC2
YTHDF1
YTHDF2
YTHDF3
YWHAZ
YY1
YY1AP1
YY2
ZAK
ZBTB10
ZBTB38
ZBTB4
ZBTB44
ZC3H11A
ZC3H13
ZC3H15
ZC3H4

ZC3H7A
ZC3H7B
ZC3HAV1
ZCCHC14
ZCCHC8
ZDHHC17
ZDHHC20
ZDHHC5
ZDHHC7
ZDHHC9
ZEB1
ZEB2
ZFAND1
ZFAND3
ZFP106
ZFP64
ZFP91-CNTF
ZFP91
ZFR
ZFYVE16
ZFYVE9
ZKSCAN1
ZMIZ1
ZMPSTE24
ZMYM2
ZMYM3
ZMYM4
ZMYND11
ZNF12
ZNF146
ZNF148
ZNF185
ZNF217
ZNF24
ZNF25
ZNF280D
ZNF282
ZNF302
ZNF331
ZNF33A
ZNF33B
ZNF362
ZNF395
ZNF496
ZNF512
ZNF592
ZNF638
ZNF639

ZNF664
ZNF704
ZNF740
ZNF770
ZNFX1
ZRANB1
ZRANB2
ZW10
ZXDC
ZYG11B
ZZEF1
ZZZ3

Supplementary Table 3. Details of the antibodies used for immunoblotting in this study.

Antibody	Company	Cat No	Dilution	Molecular Weight (Kda)
p-Ser2-RNAPII	Active Motif	61083	1;500	240
p-Ser5-RNAPII	Active Motif	61085	1;1000	240
RNAPII	Active Motif	61667	1;1000	240
Cyclin T1	Santa cruz	sc10750	1;2000	81
H3k36me3	Abcam	ab9050	1;2000	17
Pan-Acetyl-H3	Active Motif	39140	1;2000	17
H3	Cell Signaling	4499	1;2000	17
β-Actin	Cell Signaling	12620S	1;5000	45
Caspase 8	Cell Signaling	5125s	1;1000	45
IFNAR1	Abcam	ab45172	1;500	130
IFNAR2	Abcam	ab193410	1;500	58
IFNGR1	Abcam	ab134070	1;500	54
IFNGR2	Abcam	ab171081	1;500	38
JAK1	Cell Signaling	3332S	1;500	130
JAK2	Cell Signaling	3230	1;500	125
IKK-α (CHUK)	Cell Signaling	2682s	1;1000	85
JNK (MAPK8/9)	Cell Signaling	9252s	1;1000	46,54
p-STAT1	Cell Signaling	7649s	1;1000	91
STAT1	Cell Signaling	9175s	1;1000	91
p-NF-κB	Cell Signaling	3033s	1;1000	65
NF-κB	Cell Signaling	8242s	1;1000	65
MHC-I (Purified anti-human HLA-A,B,C Antibody)	Biolegend	311402	1;1000	45
β2m	Abcam	ab75853	1;1000	14
GAPDH	Cell Signaling	5174s	1;1000	37
H2-K^D	Biolegend	116601	1;500	41
Cleaved Caspase 8	Cell Signaling	9496	1;500	18, 41, 43
Anti-Rat	Cell Signaling	7077S	1;5000	
Anti-Rabit	Cell Signaling	7074	1;5000	
Anti-Mouse	Cell Signaling	7076	1;5000	
